# Quantifying and Modeling the Acquisition and Retention of Lumpy Skin Disease Virus by Hematophagus Insects Reveals Clinically but Not Subclinically Affected Cattle Are Promoters of Viral Transmission and Key Targets for Control of Disease Outbreaks

**DOI:** 10.1128/JVI.02239-20

**Published:** 2021-04-12

**Authors:** Beatriz Sanz-Bernardo, Ismar R. Haga, Najith Wijesiriwardana, Sanjay Basu, Will Larner, Adriana V. Diaz, Zoë Langlands, Eric Denison, Joanne Stoner, Mia White, Christopher Sanders, Philippa C. Hawes, Anthony J. Wilson, John Atkinson, Carrie Batten, Luke Alphey, Karin E. Darpel, Simon Gubbins, Philippa M. Beard

**Affiliations:** a The Pirbright Institute, Pirbright, Surrey, United Kingdom; b The Roslin Institute, Edinburgh, United Kingdom; c MSD Animal Health, Walton, Milton Keynes, United Kingdom; University of Illinois at Urbana Champaign

**Keywords:** poxvirus, lumpy skin disease virus, vector transmission

## Abstract

Lumpy skin disease virus (LSDV) is a vector-transmitted poxvirus that causes disease in cattle. Vector species involved in LSDV transmission and their ability to acquire and transmit the virus are poorly characterized. Using a highly representative bovine experimental model of lumpy skin disease, we fed four model vector species (Aedes aegypti, Culex quinquefasciatus, Stomoxys calcitrans, and Culicoides nubeculosus) on LSDV-inoculated cattle in order to examine their acquisition and retention of LSDV. Subclinical disease was a more common outcome than clinical disease in the inoculated cattle. Importantly, the probability of vectors acquiring LSDV from a subclinical animal (0.006) was very low compared with that from a clinical animal (0.23), meaning an insect feeding on a subclinical animal was 97% less likely to acquire LSDV than one feeding on a clinical animal. All four potential vector species studied acquired LSDV from the host at a similar rate, but Aedes aegypti and *Stomoxys calcitrans* retained the virus for a longer time, up to 8 days. There was no evidence of virus replication in the vector, consistent with mechanical rather than biological transmission. The parameters obtained in this study were combined with data from studies of LSDV transmission and vector life history parameters to determine the basic reproduction number of LSDV in cattle mediated by each of the model species. This reproduction number was highest for *Stomoxys calcitrans* (19.1), followed by *C. nubeculosus* (7.1) and *Ae. aegypti* (2.4), indicating that these three species are potentially efficient transmitters of LSDV; this information can be used to inform LSD control programs.

**IMPORTANCE** Lumpy skin disease virus (LSDV) causes a severe systemic disease characterized by cutaneous nodules in cattle. LSDV is a rapidly emerging pathogen, having spread since 2012 into Europe and Russia and across Asia. The vector-borne nature of LSDV transmission is believed to have promoted this rapid geographic spread of the virus; however, a lack of quantitative evidence about LSDV transmission has hampered effective control of the disease during the current epidemic. Our research shows subclinical cattle play little part in virus transmission relative to clinical cattle and reveals a low probability of virus acquisition by insects at the preclinical stage. We have also calculated the reproductive number of different insect species, therefore identifying efficient transmitters of LSDV. This information is of utmost importance, as it will help to define epidemiological control measures during LSDV epidemics and of particular consequence in resource-poor regions where LSD vaccination may be less than adequate.

## Abstract (precis)

Lumpy skin disease virus (LSDV) causes a severe systemic disease characterized by cutaneous nodules in cattle. LSDV is a rapidly emerging pathogen, having spread since 2012 into Europe and Russia and across Asia.

## INTRODUCTION

Lumpy skin disease virus (LSDV) is a large DNA virus of the family *Poxviridae* and the etiological agent for lumpy skin disease (LSD) in cattle. LSDV is a rapidly emerging pathogen that is believed to be mechanically transmitted by arthropod vectors. First described in Zambia in cattle in 1929, LSDV subsequently spread throughout Africa and into the Middle East (1). In the past decade, the virus has increased its geographical coverage substantially, entering and spreading within Europe and Asia, including Russia, India, Bangladesh, Taiwan, and China (2–8). LSD is characterized by fever, weight loss, and prominent multifocal necrotizing cutaneous lesions (9) and affects cattle of all ages (10). Morbidity in disease outbreaks ranges from 5% to 26% and mortality from 0.03% to 2% (2–4, 11–13). Control measures include vaccination, quarantine, and partial or complete culling of infected herds. The LSD outbreaks and subsequent control measures cause significant negative economic and welfare effects, resulting in food insecurity for affected communities in endemic (14–16) and epidemic (17) situations.

To date, the mode of LSDV arthropod transmission has been assumed to be mechanical, as no evidence of active virus replication in insects or ticks has been found (18). This mechanical arthropod-borne spread is believed to have enabled the rapid geographic expansion of LSDV; however, fundamental yet crucial answers to questions, such as the species of arthropods responsible and the infectious period of LSDV-infected cattle, remain unknown. This incomplete knowledge of LSDV transmission has impeded the implementation of targeted and evidence-based control measures.

Hematophagus dipterans (referred to in this work as “blood-feeding insects”), particularly *Stomoxys calcitrans*, have been associated with outbreaks of LSDV (7, 19–21), mainly based on inference of transmission patterns and insect ecological parameters. In addition, proof-of-principle experimental transmission of LSDV from affected to naive animals (defined by the presence of clinical disease and/or detection of systemic LSDV antigen and/or capripoxvirus-specific antibodies) has been demonstrated via the mosquito Aedes aegypti (22); the ticks Rhipicephalus appendiculatus (23–25), Rhipicephalus decoloratus (26), and Amblyomma hebraeum (27); the stable fly Stomoxys calcitrans; and horseflies *Haematopota* spp. and other *Stomoxys* species (28, 29). LSDV DNA has also been detected in other species after feeding on infected cattle or an infectious blood meal (Culex quinquefasciatus, Anopheles stephensi, and Culicoides nubeculosus) (30) or in field-caught pools (Culicoides punctatus) (4). However, transmission of LSDV to susceptible animals has not been confirmed for these species.

Overall, these studies provide growing evidence of the potential participation of different arthropods in the transmission of the LSDV. Unfortunately, previous studies had design limitations, including reduced number of donor cattle and limited times postinfection, the use of virus-spiked blood meals, and/or reduced number of insects assayed. As a consequence, the results obtained do not fulfil quantitative requirements to assess the risk of transmission. This shortcoming is demonstrated by large uncertainty in parameter estimates. Furthermore, the vital knowledge gap of understanding how efficient each vector is at contributing epidemiologically to the transmission of LSDV remains.

LSDV can be detected in skin lesions; blood (primarily in peripheral blood mononuclear cells); and nasal, oral, and ocular secretions of infected cattle (28, 31, 32). Viremia is considered of short duration and at a relatively low level, although the virus can survive for longer periods of time in skin lesions (31). LSDV has also been detected in seminal fluid of diseased bulls (33), making venereal transmission a possibility (34–36). Subclinical infections (detection of LSDV in animals without cutaneous lesions) (3, 28, 32) and resistance to LSDV (absence of LSDV and cutaneous lesions following experimental challenge) have been reported, but both are poorly documented. The contribution of subclinical LSD to the transmission of the virus is unclear and a topic of controversy when implementing control measures, such as whole-herd culling (including asymptomatic animals), particularly when morbidity is low (37, 38).

In this study, we used a highly relevant experimental LSD infection model in the natural cattle host and four representative blood-feeding insect species previously reported to be capable of acquiring LSDV (*S. calcitrans*, *Ae. aegypti*, *Cx. quinquefasciatus*, and *C. nubeculosus*). We obtained quantitative data describing critical, biologically relevant parameters of the mechanical transmission of LSDV in unprecedented detail. These transmission parameters subsequently enabled advanced mathematical modeling to understand the risk of transmission of the virus from experimentally infected cattle to each vector insect species. Furthermore, our *in vivo* transmission studies provided much-needed evidence that subclinically infected cattle do not contribute efficiently to virus acquisition by a blood-feeding insect and also further defined the infectious period of the cattle host. In combination with published data on insect ecology parameters and the feasibility of LSDV transmission, our results ultimately allowed the determination of the basic reproduction number for each insect vector species. These studies highlight the powerful combination of natural host infection/transmission studies and subsequent mathematical modeling to enable maximum relevance and transferability of data to the in-field situation and direct applicability to improve control measures.

## RESULTS

### Experimental infection of calves with LSDV.

**FIG 1:**
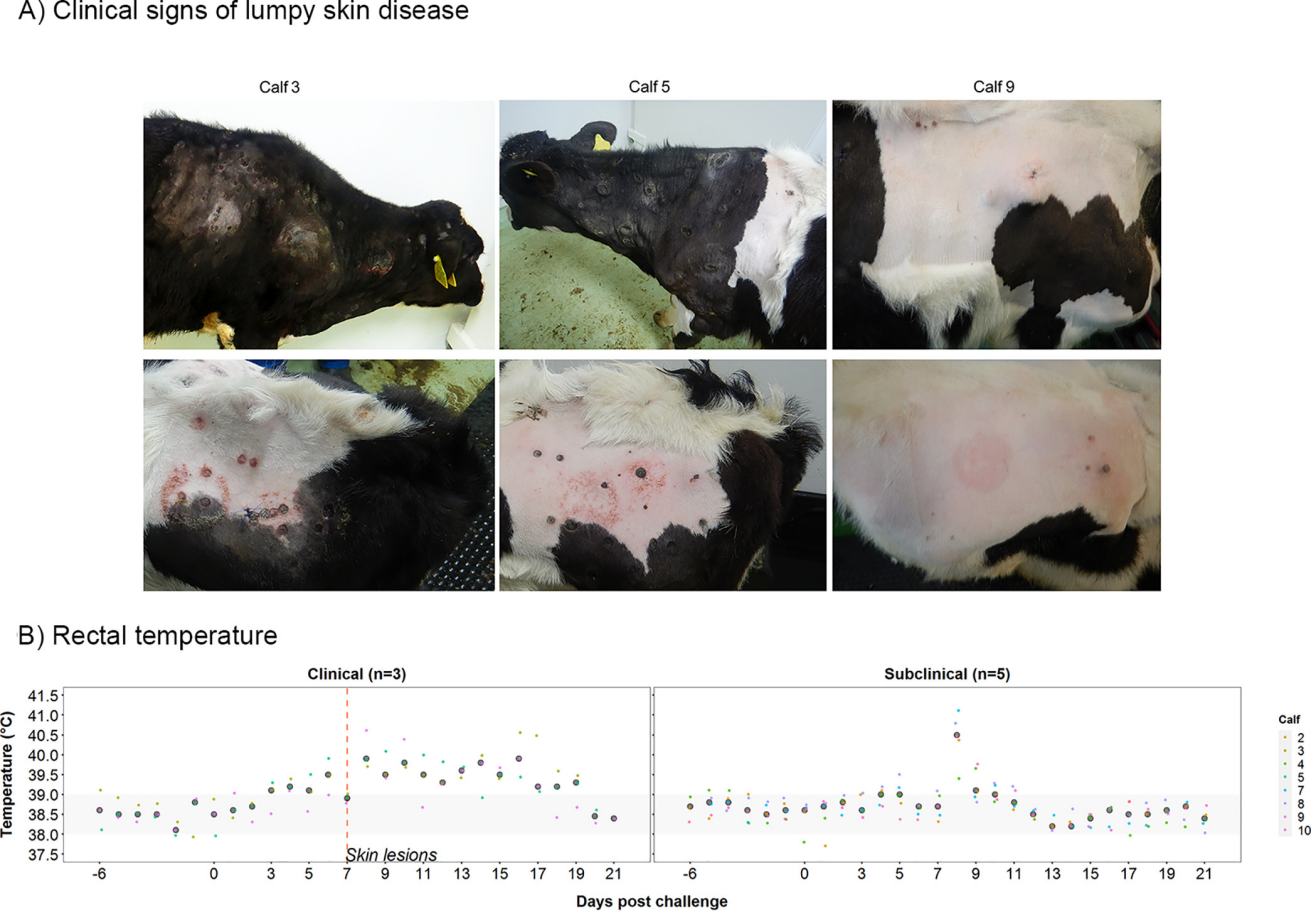
Clinical characterization of cattle experimentally challenged with lumpy skin disease virus. (A) Gross pathology of experimental LSD in cattle, characterized by severe multifocal necrotizing dermatitis. (B) Rectal temperature (°C) for clinical (left) and subclinical (right) calves.

**(i) Experimental inoculation of calves with LSDV results in clinical and subclinical disease.** Eight calves were challenged by intravenous and intradermal inoculation of LSDV in order to act as donors on which blood-feeding insects could feed. The clinical and pathological findings have been described previously (9), and they resemble those of naturally infected cattle (2, 4, 8, 11, 12, 37). Three calves (calves 3, 5, and 9) developed lumpy skin disease, characterized by severe multifocal dermatitis with necrotizing fibrinoid vasculitis consistent with field reports of LSD (Fig. 1A). The cutaneous lesions initially appeared in close proximity to the inoculation site at 5 days postchallenge (dpc) for calves 5 and 9 and at distant sites in all three clinical calves at 7 dpc. The five remaining calves (calves 2, 4, 7, 8, and 10) did not develop lesions other than at the inoculation sites. All eight inoculated calves developed a fever which was more prolonged in calves with clinical signs (Fig. 1B). Superficial lymph nodes, predominantly the superficial cervical lymph node, were enlarged in both groups starting between 2 and 5 dpc, with larger lymph nodes present in clinical than in subclinical calves. Two noninoculated in-contact calves (calves 1 and 6) were included in the study and did not develop any clinical signs or lesions consistent with LSD.

**FIG 2:**
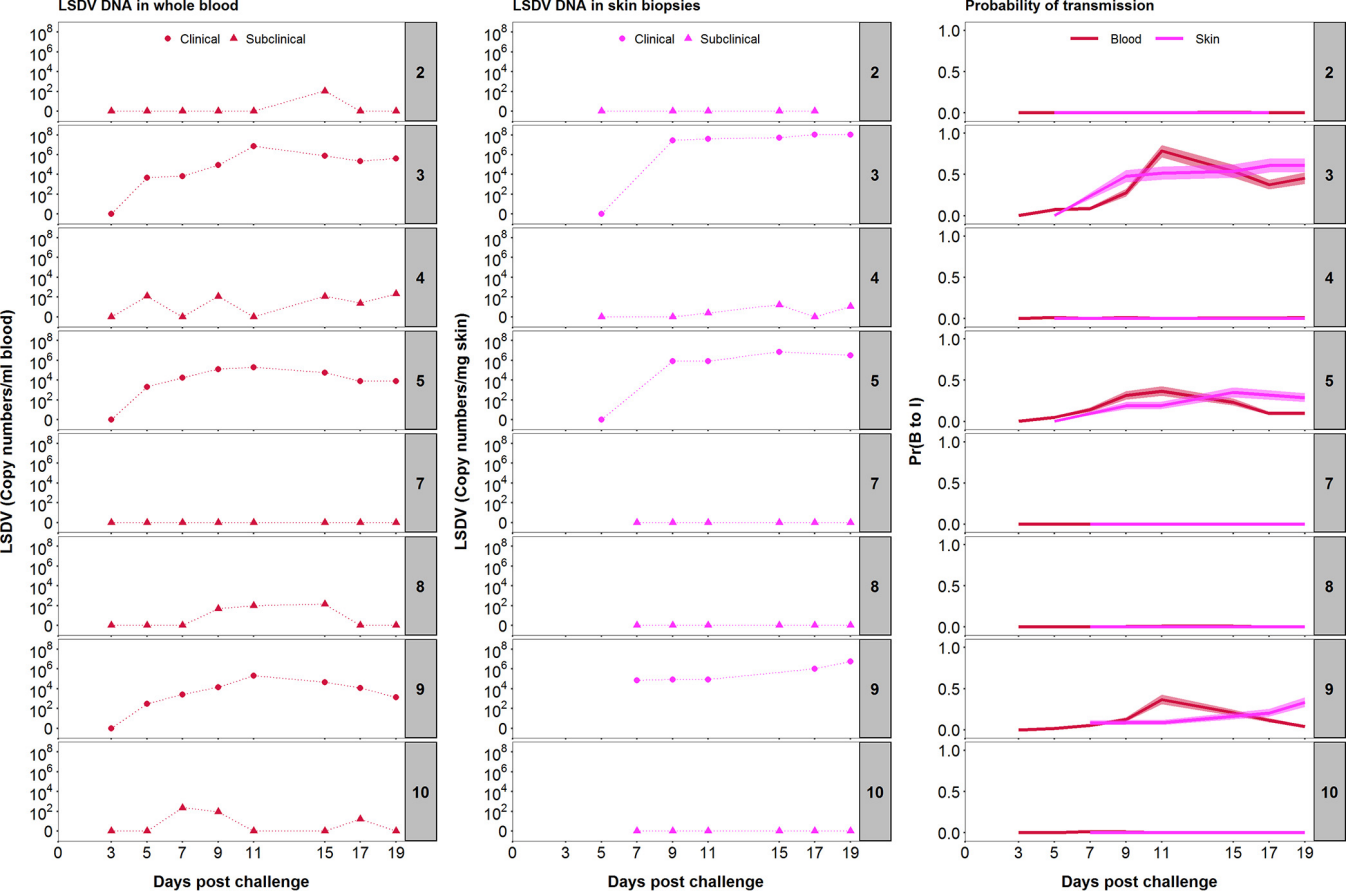
LSDV inoculation results of eight calves in a spectrum of infectiousness. Levels of viral DNA in blood (log_10_ copies/ml; first column) and skin (log_10_ copies/mg; second column) of the inoculated calves at different days postchallenge were quantified by qPCR. Based on the viral DNA levels in blood (red) or skin (magenta), the corresponding probability of transmission from bovine to insect (“infectiousness”) was calculated using a dose-response relationship (third column). Lines and shading show the posterior median and 95% credible intervals for the probability, respectively. The numbers to the right of each panel indicate the serial numbers of the calves.

**(ii) LSDV DNA can be detected in blood and skin of clinical and subclinical calves.** In the three clinically affected calves, viral DNA was first detected in the blood by quantitative PCR (qPCR) at 5 dpc and remained detectable in all subsequent blood samples (up to 19 dpc). Peak viral DNA levels in the blood (6.9, 5.3, and 5.3 log_10_ copies/ml in calves 3, 5, and 9, respectively) were reached at 11 dpc (Fig. 2). In contrast, viral DNA was detected only intermittently in the blood of four (out of five) subclinically infected calves between 5 dpc and 19 dpc. In addition, genome copy numbers were lower (median, 2.1 log_10_ copies/ml; range, 1.2 to 2.4 log_10_ copies/ml) in subclinically infected calves than those in clinically affected calves (Fig. 2). Although negative for LSDV in whole blood, the peripheral blood mononuclear cell (PBMC) fraction of calf 7 was positive for viral DNA on days 7, 9, and 19 postchallenge (see Data Set S1 in the supplemental material). These results indicate that clinical calves had more viral DNA present in the blood and for longer than that of subclinical calves. However, LSDV DNA could be detected at least once in all eight challenged animals between 5 and 19 dpc.

Skin biopsy samples of cutaneous lesions taken at 7 dpc (calf 9) or 9 dpc (calves 3 and 5) contained abundant viral genomes as measured by qPCR (Fig. 2). Viral DNA was detected in all subsequent biopsy samples, with the quantities detected remaining at an approximately constant level for the duration of the experiment (Fig. 2). The amount of viral DNA present in the skin lesions varied between the three clinical calves in an analogous fashion to the viral DNA in blood, with the highest concentration of viral DNA detected in skin lesions of calf 3 and least in calf 9 (Fig. 2). The peak level of viral DNA in skin was reached after the peak level of viral DNA in blood in all three calves (Fig. 2). Viral DNA was detected at three time points in biopsy samples of normal skin from one subclinical calf (calf 4) at a lower copy number than in the clinically affected animals; skin biopsy samples from the other subclinical animals (calves 2, 7, 8, and 10) were all negative for LSDV DNA (Fig. 2).

**FIG 3:**
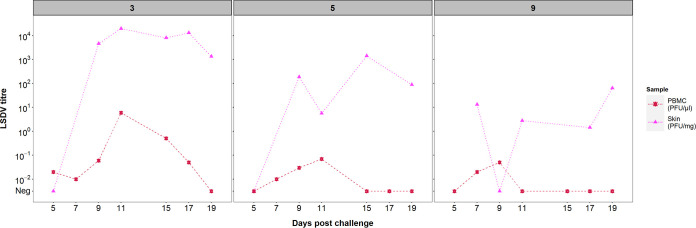
LSDV titers vary between three clinical animals but are consistently higher in the skin than in the blood. Levels of infectious lumpy skin disease virus (LSDV) in skin biopsy samples (PFU/mg of skin) (magenta triangles) and peripheral blood mononuclear cell (PBMC) fractions (PFU/μl suspension) (red stars) were quantified by plating onto MDBK cells. Generalized skin lesions were first noted in all three animals at 7 days postchallenge. The numbers above each panel indicate the serial numbers of the calves.

**(iii) Infectious LSDV is present in larger quantities in the skin than in the blood.** Both skin homogenate and PBMC suspension collected between 5 and 19 dpc from clinical calves were titrated to determine the quantity of live virus in these tissues. Although units of measurement are not directly comparable between sample types (i.e., skin versus PBMC), they are representative of the magnitude of exposure that hematophagous insects may encounter during feeding (i.e., mg of skin tissue and μl of blood). In all calves, the viral titer from skin homogenate was higher and more constant than from PBMC suspension (Fig. 3). Live virus was detected for six consecutive days from 5 dpc in the PBMC fraction of calf 3, whereas in calves 5 and 9, the virus was isolated only in 3 and 2 days, respectively, starting at day 7 postchallenge. In contrast, all skin samples except one taken from dermal lesions contained live LSDV with a maximum titer of 10^4.3^ PFU/mg skin, which is over 10^3^-fold greater than the maximum level of virus detected in PBMCs, emphasizing the strong cutaneous tropism of LSDV. Biopsy samples collected from normal skin of clinical calves were negative for live virus (i.e., below 10^−2^ PFU/mg) (Data Set S1) suggesting the virus is highly concentrated in the skin lesions of clinical animals. Live virus was not detected in blood or skin samples from subclinical animals (including samples which were qPCR positive).

**(iv) Humoral response to LSDV inoculation.** Serum from the three clinically affected calves contained antibodies to LSDV at 15 to 17 dpc, as determined by a commercial enzyme-linked immunosorbent assay (ELISA). By the end of the study period, all subclinical animals had also developed detectable LSDV antibodies at levels lower than those observed in the clinical animals but above those of the nonchallenged controls (data not shown). The presence of detectable levels of antibodies confirmed exposure to the virus in all eight challenged animals, although the clinical outcome of challenge varied widely between the eight calves.

### Acquisition and retention of LSDV by blood-feeding insects after feeding on donor cattle.

**FIG 4:**
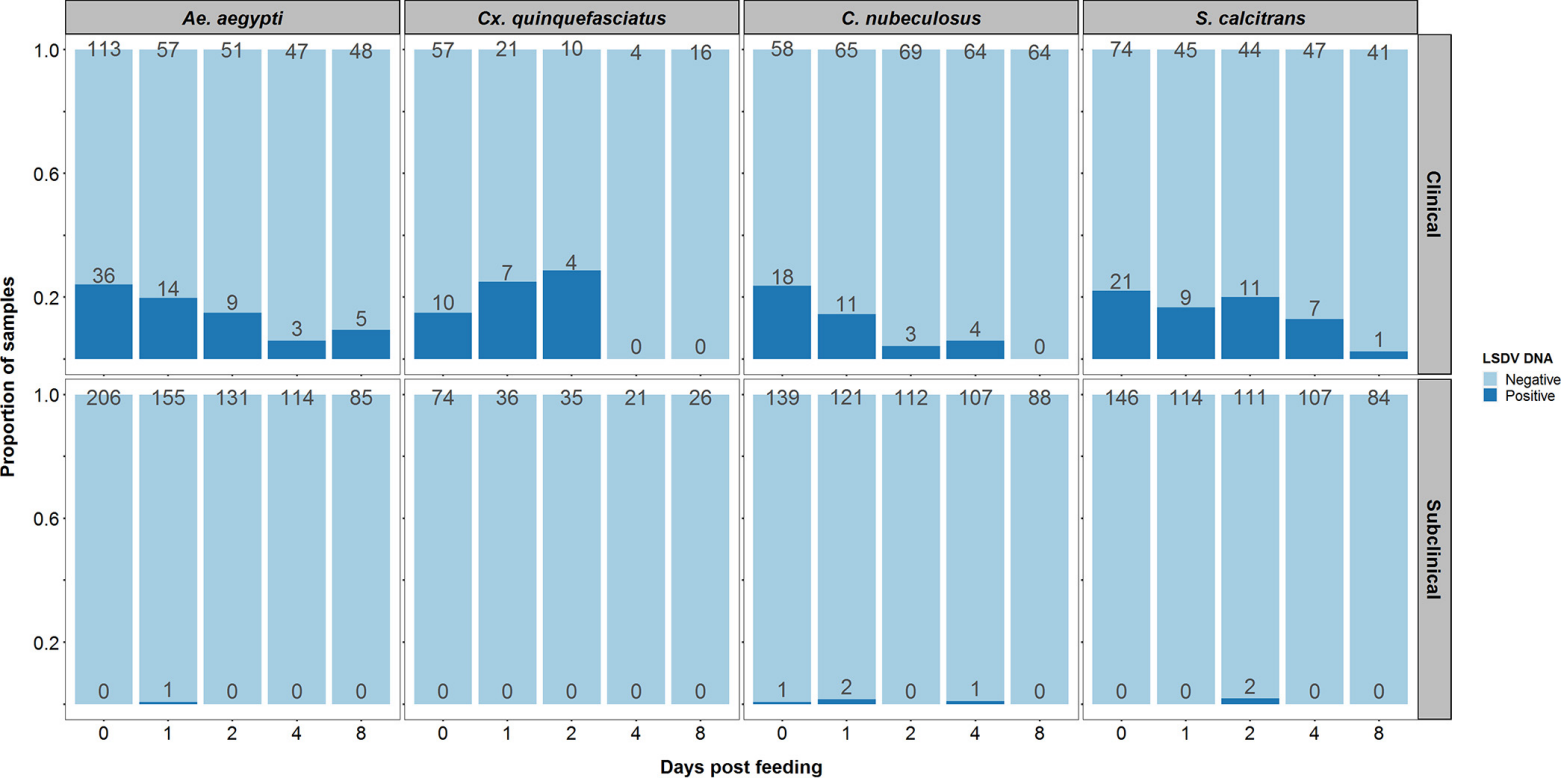
A higher proportion of insects are positive for lumpy skin disease viral DNA after feeding on a clinical animal than on a subclinical animal. The plots show the number of insects of each species tested (above each pale-blue bar) and the number of insects positive for viral DNA (above each dark-blue bar) at each day postfeeding on clinical (top) or subclinical (bottom) donor cattle.

We next studied the influence of this disease spectrum on the acquisition and retention of LSDV in blood-feeding insects. To assess the acquisition and retention of LSDV by blood-feeding insects, all eight challenged animals were exposed to two mosquito species, *Ae. aegypti* and *Cx. quinquefasciatus*; one species of biting midge, *C. nubeculosus*; and the stable fly *S. calcitrans* on days 5, 7, 9, 11, 15, 17, and 19 postchallenge. The selected species are potential mechanical vectors with different feeding mechanisms (39), covering those which will feed readily on cattle (i.e., *S. calcitrans*), as well as species models for *Culex* and *Aedes* mosquitoes (40, 41) and biting midges (42, 43) which would feed on cattle. At each time point, a pot of insects of each species (i.e., four pots in total) was placed on a separate cutaneous nodule on a clinical animal and on a corresponding area of normal skin on a subclinical animal. Blood engorgement, as a measure for detection of insect biting activity, was assessed visually. A subset of the insects from each pot was tested for the presence of LSDV DNA by qPCR at 0, 1, 2, 4, and 8 days post feeding (dpf) (Fig. 4). The smaller numbers of insects tested at the later time points reflect the lower numbers surviving for long enough to be tested.

Different models for the proportion of positive insects were compared to assess differences in (i) the probability of transmission from bovine to insect (i.e., of acquiring LSDV) among insect species and between clinical and subclinical donors, and (ii) the duration of viral retention among insect species (see Appendix). Models were compared using the deviance information criterion (DIC), with a model having a lower DIC preferred to one with a higher DIC. Positive insects were those with LSDV DNA amplification by qPCR.

### (i) Probability of transmission from bovine to insect.

**FIG 5:**
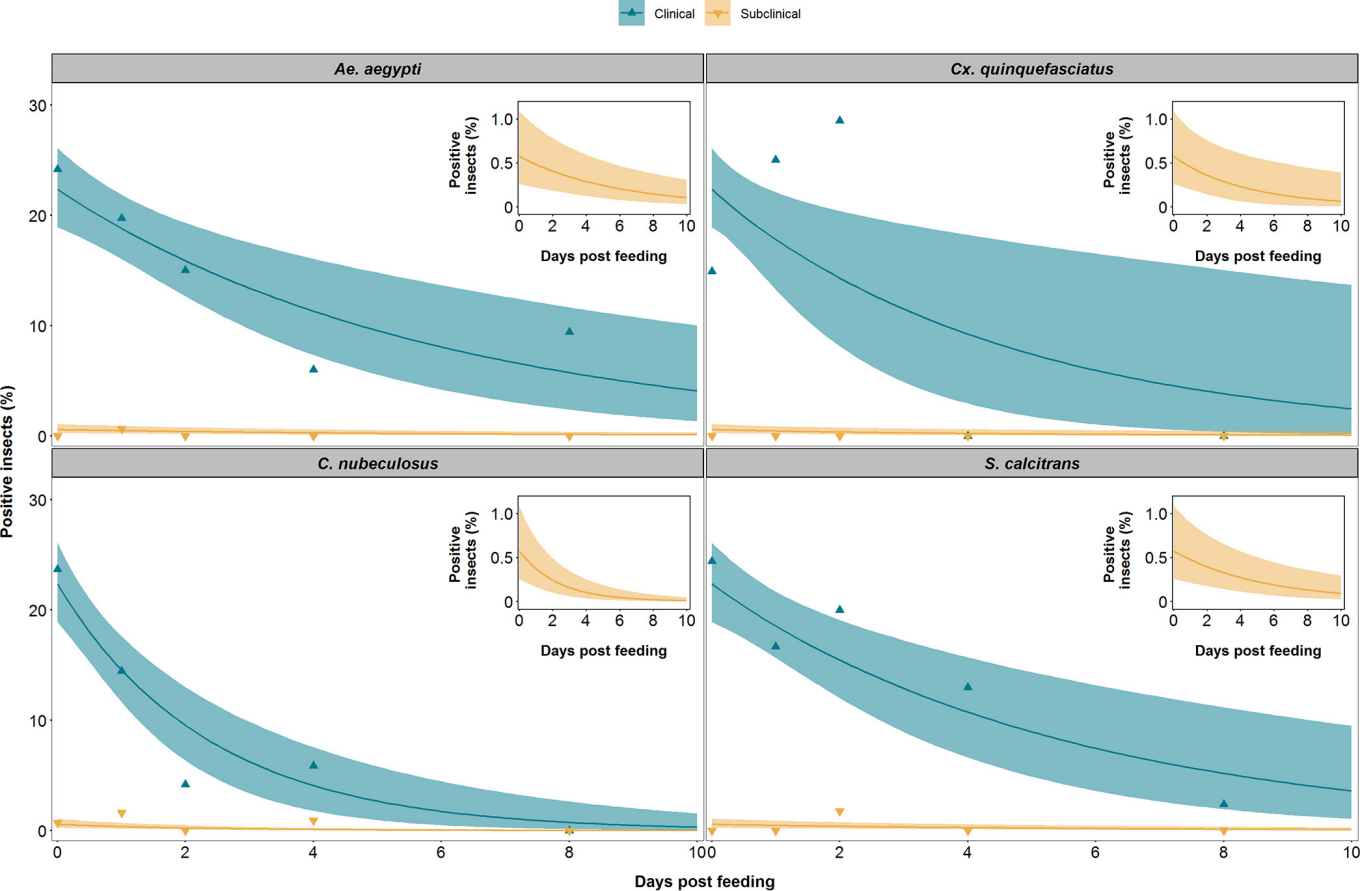
LSDV is retained in blood-feeding insects for up to 8 days postfeeding. The proportion of blood-feeding insects positive for lumpy skin disease viral DNA after feeding on a clinical (green) or subclinically (yellow) animal is shown for the four species of insects, namely, Aedes aegypti, Culex quinquefasciatus, *Culicoides nubeculosus*, and *Stomoxys calcitrans*. Each plot shows the observed proportion of positive insects (triangles) and the expected proportion of positive insects (posterior median [line], and 2.5th and 97.5th percentiles of the posterior distribution [shading]). The inset shows the expected proportion of positive insects after feeding on a subclinical animal using a graph with an expanded *y* axis.

A total of 3,178 insects were fed on the 8 donor calves (over 7 feeding sessions), of which 180 were positive for viral DNA. A higher proportion of insects were positive after feeding on a clinical donor (173 out of 1,159) than after feeding on a subclinical donor (7 out of 2,019) (Fig. 4). Comparing the proportion of positive insects for each species after feeding in clinical and subclinical calves (Fig. 5) revealed that the probability of transmission from bovine to insect (i.e., of acquiring LSDV) does not differ among the four insect species but that this probability does differ between clinical and subclinical donors (see Appendix). For a clinical donor, the probability of transmission from bovine to insect was estimated (posterior median) to be 0.22, while for a subclinical donor, it was estimated to be 0.006 (Table 1). This result means that an insect feeding on a subclinical animal is 97% less likely to acquire LSDV than an insect feeding on a clinical one (Table 1; Fig. 5).

**TABLE 1:** Parameters for transmission of LSDV by four species of biting insects

Parameter	Estimate^*a*^
Probability of transmission from bovine to insect^*b*^
Clinical donor (*β)*	0.22 (0.19, 0.26)
Subclinical donor (*ρβ)*	0.006 (0.003, 0.011)
Relative risk of transmission from a subclinical compared with clinical bovine^*b*^ (*ρ)*	0.03 (0.01, 0.05)
Virus inactivation rate (/day) (*γ)*
*Ae. aegypti*	0.17 (0.07, 0.29)
*Cx. quinquefasciatus*	0.22 (0.05, 0.51)
*C. nubeculosus*	0.42 (0.26, 0.64)
*S. calcitrans*	0.18 (0.08, 0.31)
Mean duration of virus retention (days) (1/*γ)*
*Ae. aegypti*	5.9 (3.5, 13.4)
*Cx. quinquefasciatus*	4.5 (2.0, 22.0)
*C. nubeculosus*	2.4 (1.6, 3.9)
*S. calcitrans*	5.5 (3.2, 12.3)
Probability of transmission from insect to bovine (*b)*
*Ae. aegypti*	0.56 (0.11, 0.98)
*Cx. quinquefasciatus*	0.11 (0.004, 0.73)
*C. nubeculosus*	0.19 (0.007, 0.91)
*S. calcitrans*	0.05 (0.02, 0.15)
Basic reproduction no. (*R*_0_)
*Ae. aegypti*	2.41 (0.50, 5.22)
*Cx. quinquefasciatus*	0.55 (0.06, 2.37)
*C. nubeculosus*	7.09 (0.24, 37.10)
*S. calcitrans*	19.09 (2.73, 57.03)

a Posterior median (95% credible interval).

b Parameter does not differ among species.

### (ii) Infectiousness correlates with the level of viral DNA in blood and skin.

**FIG 6:**
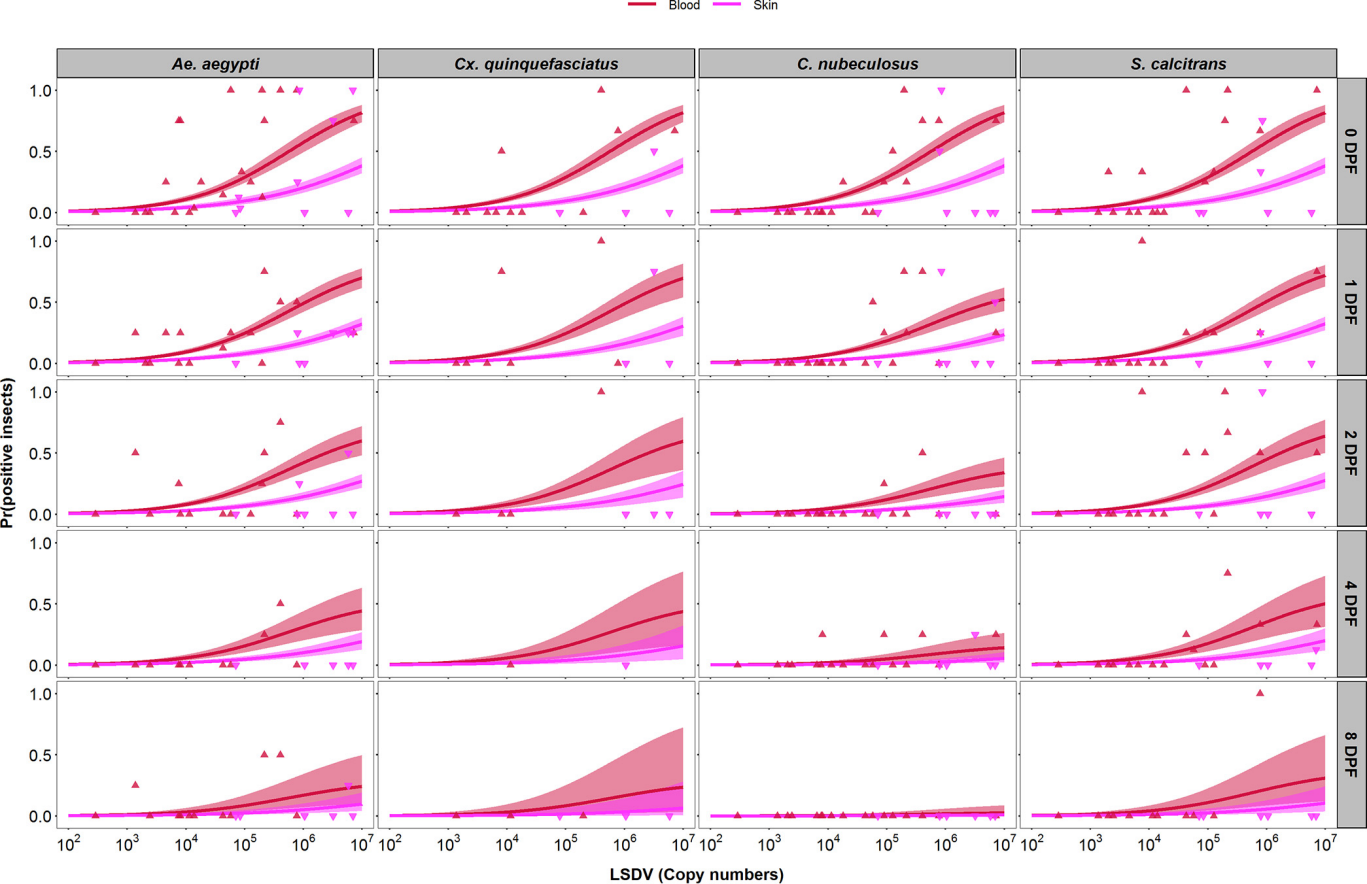
Levels of lumpy skin disease viral DNA in blood or skin are proxy measures of infectiousness. Each plot shows the dose-response relationship between the probability of an insect being positive for lumpy skin disease virus (LSDV) DNA and the level of viral DNA in the blood (log_10_ copies/ml; red) or skin (log_10_ copies/mg; magenta) of the calf on which they fed. Four species of insects, namely, Aedes aegypti (first column), Culex quinquefasciatus (second column), *Culicoides nubeculosus* (third column), or *Stomoxys calcitrans* (fourth column), were tested at 0, 1, 2, 4, and 8 days postfeeding (rows). Plots show the observed proportion of positive insects (blood, red up triangles; skin, magenta down triangles) and the estimated probability of an insect being positive (posterior median [line] and 2.5th and 97.5th percentiles of the posterior distribution [shading: blood, red; skin, magenta]).

The relationship between the level of viral DNA in the skin or blood of a calf and the proportion of virus-positive insects resulting from a feeding session was examined. For each feeding session that took place on the three clinical calves, the proportion of insects containing viral DNA postfeeding was calculated and compared with the viral DNA copy number present in both the blood sample and the skin biopsy sample taken from the calf on that day (Fig. 6). This comparison revealed a dose-response relationship between the levels of viral DNA in skin and blood and the probability of transmission from bovine to insect (or “donor infectiousness”). Furthermore, this relationship was the same for all four insect species (see Appendix), irrespective of their different feeding mechanisms. The relationship differed between levels of viral DNA in blood and skin (Table 2; Fig. 6), with the probability of transmission being higher when using the level of viral DNA in blood than that in skin (Fig. 6). The fits of the models using levels of viral DNA in blood or skin were similar, suggesting that both are acceptable proxy measures for infectiousness of the donor.

**TABLE 2:** Parameters for the dose-response relationship between levels of viral DNA

Dose-response parameters	Estimated level of viral DNA in:^*a*^	
	Blood	Skin
Intercept (*d*_0_)	−6.89 (−7.74, −6.11)	−6.70 (−7.81, −5.76)
Slope (*d*_1_)	1.20 (1.03, 1.38)	0.89 (0.75, 1.06)

a Posterior median (95% credible interval).

Combining the dose-response relationship (Fig. 6) with the time course for levels of viral DNA in blood or skin for each calf (Fig. 2) shows how the infectiousness of an animal changes over time and how it varies among animals (Fig. 2, right-hand column). This result highlights the very low probability of transmission from bovine to insect (<0.01 at all time points; cf. estimate in Table 1) for calves which were only subclinically infected. In addition, for those calves which did develop clinical signs, the probability of transmission from bovine to insect was much lower before the onset of clinical signs (around 7 dpc) than afterward. There were also differences in both the timing and level of infectiousness among the clinical calves, which is a consequence of the underlying differences in viral dynamics in each animal.

### (iii) Duration of LSDV retention.

Viral DNA was detected in *Ae. aegypti* and *S. calcitrans* up to 8 dpf, in *C. nubeculosus* up to 4 dpf, and in *Cx. quinquefasciatus* up to 2 dpf (Fig. 4). However, few *Cx. quinquefasciatus* mosquitoes survived to 4 or 8 dpf (Fig. 4), resulting in uncertainty about the duration of retention in this species (Fig. 5 and 6). The mean duration of viral retention differed among the four insect species in the present study (Fig. 5; see Appendix), being the longest for *Ae. aegypti* (5.9 days) and *S. calcitrans* (5.5 days), followed by *Cx. quinquefasciatus* (4.5 days), and *C. nubeculosus* (2.4 days) (Fig. 5; Table 1). The corresponding virus inactivation rate (i.e., the reciprocal of the mean duration of retention) was 0.17/day for *Ae. aegypti*, 0.18/day for *S. calcitrans*, 0.22/day for *Cx. quinquefasciatus*, and 0.42/day for *C. nubeculosus* (Table 1).

### (iv) Levels of retained LSDV.

**FIG 7:**
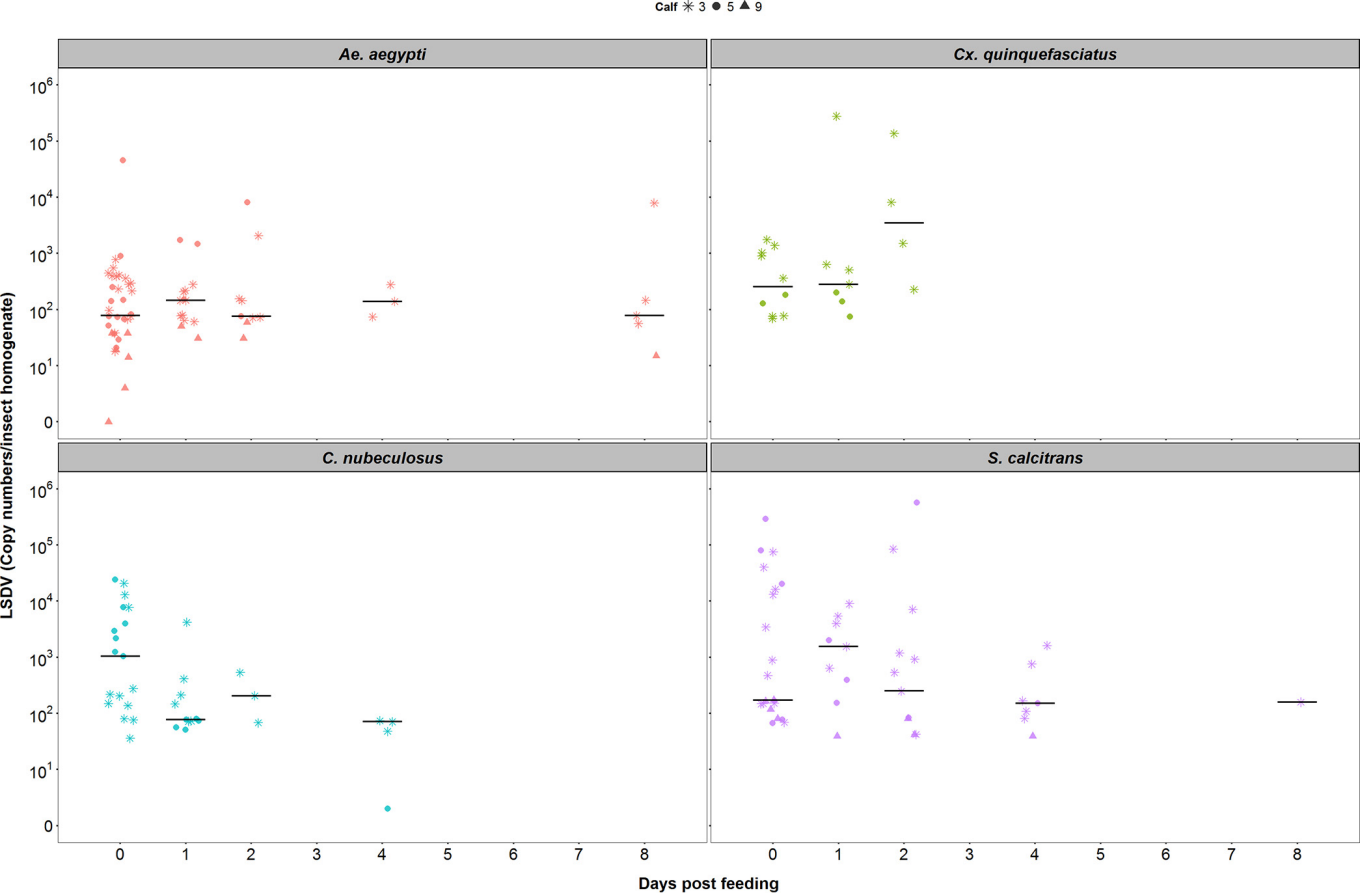
The median amount of lumpy skin disease viral DNA in homogenized whole insects was the same over time postfeeding in three out of four species tested. Each plot shows the quantity of virus in individual insects (symbols) and the median (black horizontal bars). The different symbols indicate the calf on which an insect fed.

The median amount of viral DNA in homogenized whole insects was the same when tested at different days postfeeding for the following three (out of the four) species: *Ae. aegypti* (Kruskal-Wallis test, χ^2^ = 0.98; df = 4, *P *= 0.91), *Cx. quinquefasciatus* (Kruskal-Wallis test, χ^2^ = 3.62, df = 2, *P *= 0.16), and *S. calcitrans* (Kruskal-Wallis test: χ^2^ = 2.74, df = 4, *P *= 0.60) (Fig. 7). However, the median level of viral DNA was lower for individual *C. nubeculosus* tested at later times postfeeding (Kruskal-Wallis test, χ^2^ = 10.8, df = 3, *P *= 0.01) (Fig. 7). These results are consistent with a mechanical rather than a biological form of vector transmission.

### Probability of transmission from insect to bovine.

Three previous studies have investigated the transmission of LSDV from insects to cattle, where insects of species included in the present study were allowed to feed on an infected donor and were subsequently allowed to refeed on a naive recipient (22, 28, 30). The number of positive insects refeeding was not determined in these studies. By combining LSDV acquisition and retention results of the present study with challenge outcomes of the aforementioned studies (i.e., whether or not transmission occurred), it is possible to estimate the probability of transmission from insect to bovine. This probability was highest for *Ae. aegypti* (0.56), intermediate for *C. nubeculosus* (0.19) and *Cx. quinquefasciatus* (0.11), and lowest for *S. calcitrans* (0.05) (Table 1). However, there is considerable uncertainty in the estimates for all species, but especially for *Ae. aegypti*, *C. nubeculosus*, and *Cx. quinquefasciatus* (Table 1), which makes it difficult to compare estimates across species.

### Basic reproduction number for LSDV.

**FIG 8:**
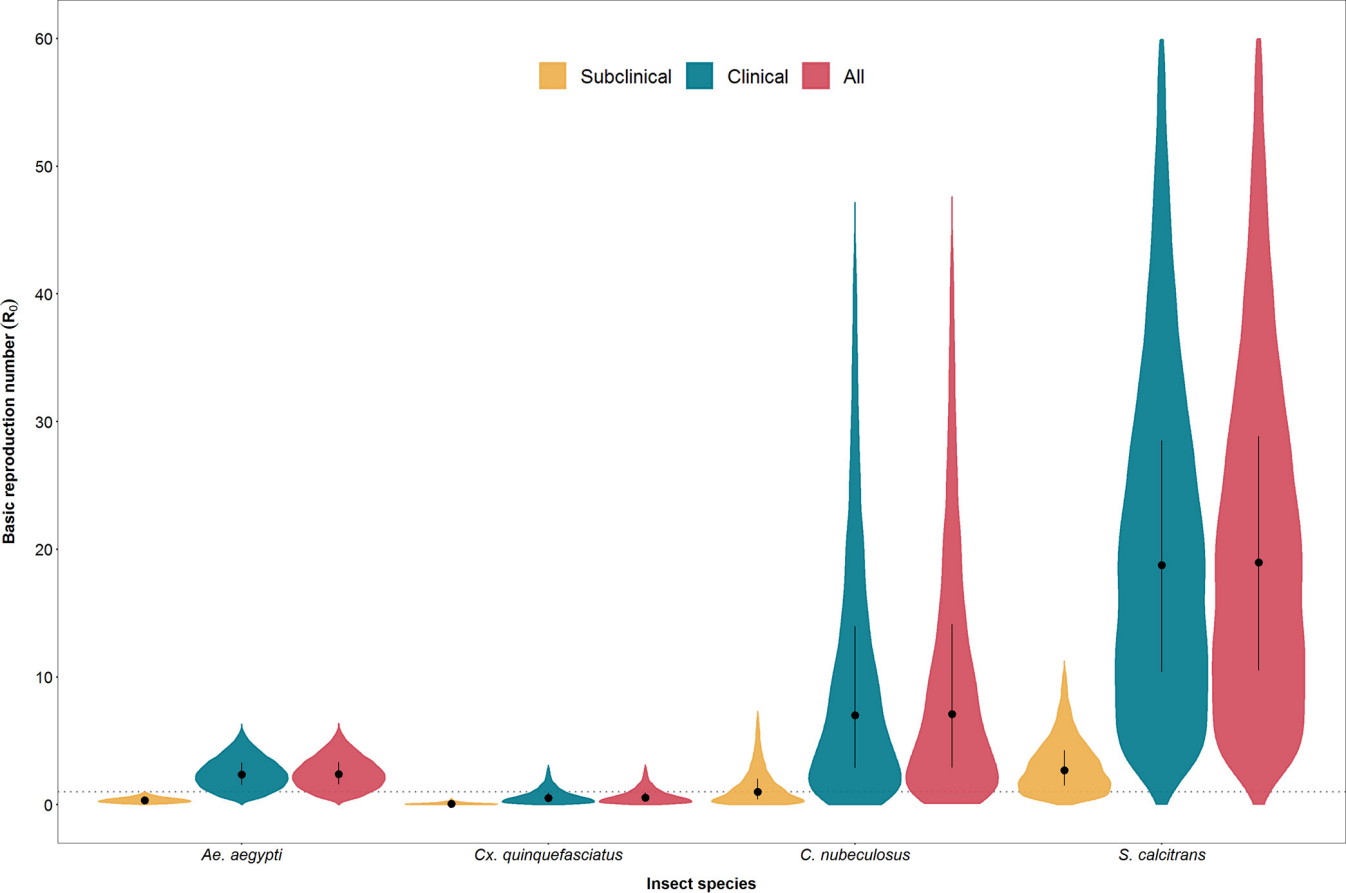
Basic reproduction number (*R*_0_) for lumpy skin disease virus (LSDV) in calves when transmitted by Aedes aegypti, Culex quinquefasciatus, *Culicoides nubeculosus*, or *Stomoxys calcitrans*. For each species, *R*_0_ was calculated for subclinical calves only (yellow), clinical calves only (green), and both combined (red). Violin plots show the posterior median (black circle), interquartile range (black vertical line), and density (shape) for *R*_0_ based on replicated Latin hypercube sampling (100 replicates with the range for each parameter subdivided into 100 steps).

The basic reproduction number (*R*_0_) is defined as “the average number of secondary cases caused by an average primary case in an entirely susceptible population” (44). For LSDV, *R*_0_ combines the parameters related to transmission (Table 1) with those related to vector life history (i.e., biting rate, vector to host ratio, and vector mortality rate) (see Table 1 in reference 45) to provide an overall picture of the risk of transmission by the four insect species (45). The basic reproduction number was estimated to be highest for *S. calcitrans* (median *R*_0_, 19.1) (Table 1; Fig. 8), indicating that this species is likely to be the most efficient vector of LSDV and would be able to cause substantial outbreaks if it were the sole vector in a region. Both *C. nubeculosus* (median *R*_0_, 7.1) and *Ae. aegypti* (median *R*_0_, 2.4) are also potentially efficient vectors of LSDV (i.e., *R*_0_ of >1 for these species) and would be able to sustain transmission if either were the sole vector in a region. Finally, *Cx. quinquefasciatus* (median *R*_0_, 0.6) is likely to be inefficient at transmitting LSDV (Table 1; Fig. 8). It would not be able to sustain transmission on its own, but it could contribute to transmission if other vector species were also present.

Exploring the contribution of clinical and subclinical animals to the basic reproduction number for each species further emphasizes the more limited role played by subclinical animals in the transmission of LSDV (Fig. 8). For all species, the *R*_0_ for clinical animals alone is very close to that for both clinical and subclinical animals combined (Fig. 8). Moreover, the median *R*_0_ for subclinical animals alone is below one for all species, except *S. calcitrans* (Fig. 8).

The *R*_0_ values calculated from our data and previous studies provide a summary of the risk of LSDV transmission. A range of blood-feeding insects are likely to support a disease outbreak by transmitting LSDV from a clinical to a naive animal, particularly biting flies, such as *S. calcitrans*. The *R*_0_ calculations also highlight that, although there may be a significant subset of subclinical animals in an affected herd, they are likely to play at most a minor role in the transmission of the virus.

## DISCUSSION

This study describes a controlled experimental model of LSD that mimics disease features described in field outbreaks (2, 4, 8, 11, 12, 37) and other experimental models (28, 32). Inoculated calves (both clinical and subclinical) were used to measure the acquisition (transmission from bovine to insect) and retention of LSDV by four potential vector species. These data were then used to estimate the risk of transmission by these species with the aim of providing evidence with which to inform decisions during the implementation of measures to control LSDV.

In our experimental model, we observed that 37.5% of calves developed generalized LSD, and the remaining 62.5% of calves were classified as subclinical (no cutaneous nodules, positive qPCR in blood [28]). This attack rate of 0.37 is comparable to that of other experimental models with field strains of LSDV (0.57 [28] and 0.50 [32]). Reports of animals with subclinical LSD in the field are sparse, with an incidence of up to 31.3% reported (3). The high detection of subclinical infection in our study may be a result of an intense sampling protocol (compared with the limited sampling of individuals during an outbreak investigation). Further investigation of the true incidence of subclinical LSD in field studies is warranted.

Cattle experimentally infected with LSDV, including in our study, have higher concentrations of LSDV in skin lesions than in blood (Fig. 2 and 3). In clinically infected animals, we identified a relationship between the viral load in skin and blood and the proportion of insects positive for the virus, indicating both skin and blood are good predictors of the transmissibility of LSDV from donors to vector. However, our study did not extend beyond 21 days postchallenge, and this observation may be true only during the initial stage of the disease when the viremia is detectable. Donors with different disease severity and therefore different levels of infectiousness would strongly influence the proportion of vectors which acquired virus. This finding may explain the discrepancies between experimental studies which have assessed the transmission of LSDV by vectors (22, 30) when the infectiousness of the donors may have been different.

It is not clear if the source of the virus acquired by the insects is skin nodules or viremic blood or a combination of both. Given the higher concentration of virus in the skin lesions than in blood (Fig. 3), we can hypothesize that lesions are the main source of acquisition. However, 7 insects acquired LSDV from subclinical cattle (Fig. 4), so skin lesions are not the sole source of virus.

As reported in this study and others (28, 31), LSDV can be detected in the blood of cattle prior to the appearance of skin lesions at 5 to 8 dpc. However, during this time, viremia is relatively low, and in our study, few insects were positive for LSDV after feeding. This low probability of virus transmission at the preclinical stage is important information, as it provides a more accurate evaluation of the latent period of virus infection. Viremia rises and peaks after the multifocal skin lesions appear (at around 7 dpc), and this is when the probability of transmission from bovine to insect starts to increase (Fig. 2). The probability remains high while viremia is high and when skin lesions are present. The appearance of skin lesions therefore marks the start of the risk period for virus transmission, and this means that rapid diagnosis and consequent implementation of control measures should be possible and effective at limiting onward transmission (46, 47). In this study, we were able to follow the animals only for 21 days postchallenge with the last exposure of blood-feeding insects to infected calves on day 19, and thus, the period for transmission risk could not be established beyond this time point. Nevertheless, under controlled conditions (31), LSDV has been isolated up to 28 (blood) and 39 (skin) days postchallenge and detected by PCR up to 91 days postchallenge (in skin biopsy samples). Therefore, LSDV uptake by vectors may occur beyond the reported period in our study.

We found that subclinical donors were much less likely than clinical animals to transmit virus to vectors (Table 1; Fig. 4 and 5), indicating a substantially reduced role of subclinically infected animals in the transmission of LSDV. For some vector-borne diseases, such as dengue fever, and malaria, asymptomatic and preclinical individuals may be an important source of the pathogen for vectors and may help maintain the transmission cycle (48, 49). The situation with LSDV appears to be different. The viremia in subclinical animals is low, and skin lesions (representing the major viral load) are absent in these animals. Few vectors therefore acquire LSDV from subclinical cattle, and this reduces the chances of onward transmission to a susceptible host. This is the first time the relative contribution of subclinically infected cattle to onward transmission of LSDV has been quantified.

Lumpy skin disease virus can be mechanically transmitted by stable and horse flies (28, 29) and mosquitoes (22). Mechanical transmission of viruses by blood-feeding vectors can be influenced by their feeding mechanism, ecology, and biting behavior. Stable flies are aggressive feeders with a painful bite which leads to interrupted feeding and to more than one feeding event per day (50, 51). They are also known to regurgitate previous blood intakes while feeding. To penetrate the skin, stable flies rotate sharp teeth on their proboscis (5 to 8 mm long) and form a pool of blood from which they feed (39). *Culicoides* midges also disrupt the skin barrier using their proboscis (0.1 to 0.2 mm long). Midges serrate the skin using saw-like blades on their proboscis that cross over each other to produce a pool of blood (39). Biting midges generally feed less frequently than stable flies, as feeding is associated with their gonotrophic cycle (7 to 10 days; but as a temperature-dependent event, it can be as short as 2 to 3 days) (52). Mosquitoes do not produce pools of blood; instead, they penetrate the skin “surgically” searching for a capillary with their proboscis (1.5 to 2.0 mm long), accompanied by a pushing and withdrawing movement until it hits a capillary from which to withdraw blood (53). Mosquito blood feeding is also associated with their gonotrophic cycle, but multiple feedings have been reported in some species (54, 55). Despite these variations in feeding behavior, all four insect species acquire LSDV at the same rate, indicating that virus acquisition is not influenced by feeding behavior.

All four insect species in the present study were able to acquire LSDV through feeding on clinical animals and to retain virus for several days (Fig. 4 and 5). In a small proportion of *Ae. aegypti* and *S. calcitrans*, LSDV DNA was still present at 8 days postfeeding. This was the latest time we investigated; thus, longer retention cannot be ruled out. Similar to our study, Chihota and coauthors (22), identified that *Ae. aegypti* mosquitoes feeding on animals with clinical LSD were able to acquire and retain the virus for up to 6 days and that the proportion of virus-positive insects also decreased with days postfeeding. They observed similar dynamics in *Cx. quinquefasciatus* and Anopheles stephensi mosquitoes when using a membrane-feeding system with a LSDV-infected bloodmeal, but when they fed *C. nubeculosus* and *S. calcitrans* on LSDV-infected calves, they did not detect the virus beyond the day of feeding (*C. nubeculosus*) or the following day (*S. calcitrans*) (30). Since we now know that disease severity of the donor can influence the acquisition of LSDV by an insect, this could account for the lower acquisition and retention observed in the Chihota et al. study. In our work, we identified that LSDV can be retained longer than previously reported in *S. calcitrans* and *C. nubeculosus*, with a decline in virus DNA postfeeding detectable only for *C. nubeculosus*.

For LSDV, as for other chordopoxviruses, including capripoxvirus, fowlpox virus, and myxoma virus, the mode of vector-mediated transmission is assumed to be mechanical (22, 56–59). Our data and those of Chihota and coauthors (22, 30) support the theory that LSDV does not replicate in the insect (at least at detectable levels), but the retention of viral DNA in *Ae. aegypti* and *S. calcitrans* at levels similar to those acquired during feeding deserves further investigation (60).

An assessment of acquisition and retention of the LSDV genome was performed in whole insect homogenates in our study, and further investigations into the location of virus within the insects were not possible. However, an earlier study with *Ae. aegypti* (61) indicated that LSDV DNA persists longer in the head than in the thorax/abdomen. This finding is consistent with research that found myxoma virus was retained on the mouthparts of *Ae. aegypti* mosquitoes up to 28 days postfeeding (62). The mechanism by which poxviruses persist for days on the mouthparts of vectors warrants further study.

The detection of LSDV in insect vectors in our study was based on the presence of viral DNA rather than infectious virus particles. Viral titer determination of homogenates of individual insects was attempted, but we were able to detect live virus only from pooled homogenates of *S. calcitrans* and of *Ae. aegypti* (data not shown). This result suggests low numbers of infectious virions are present on each insect. In previous work, live LSDV was detected in individual *Ae. aegypti* for up to 6 days following exposure to an infectious calf (22) and live goatpox virus up to 4 days in *S. calcitrans* (59).

The aim of the present study was to use the results of feeding four model vector species on LSDV-infected cattle to estimate parameters related to transmission that was not possible with data from previous studies. Given the large number of insects fed and tested (>3,000), the resulting estimates for the probability of transmission from bovine to insect (including the relative risk of transmission from a subclinical animal and the dose response) are robust, as indicated by the narrow credible intervals for these parameters (Table 1 and 2). The estimates for the duration of virus retention (or, equivalently, the virus inactivation rate) are more uncertain (Table 1), which reflects difficulties in keeping insects alive to later days postfeeding, especially *Cx. quinquefasciatus*.

Although not assessed in the present study, we used data from previous transmission experiments (22, 28) to estimate the probability of transmission of LSDV from insect to bovine. The small number of studies (and animals in each study) mean that the estimates for this parameter are uncertain, extremely so for *Ae. aegypti*, *Cx. quinquefasciatus*, and *C. nubeculosus* (Table 1). This uncertainty is less important for *Cx. quinquefasciatus*, which is unlikely to be an important vector even if it were able to transmit LSDV efficiently, but it makes it difficult to determine whether or not *C. nubeculosus* is likely be an important vector. This is of consequence because *Culicoides* spp. are ubiquitous on cattle farms (63, 64) and would represent a major transmission risk if they proved to be efficient vectors of LSDV.

The uncertainty in the estimates for *R*_0_ also reflect the wide ranges used for the vector life history parameters for each species (see Appendix) and the difficulty in providing a value for each parameter that is applicable in all situations. In particular, vector life parameters will depend on environmental factors, including temperature, precipitation, and habitat suitability. Consequently, the basic reproduction number and, hence, the risk of transmission are unlikely to be constant over space or time but will vary both geographically and seasonally. For a more complete discussion of the role of vector life history parameters, see the earlier study by Gubbins (45).

Linking transmission experiments with mathematical modeling is an uncommon but powerful approach to create robust evidence which can inform policymakers involved in controlling the spread of infectious diseases. Here, we have used this approach to investigate the transmission of LSDV, which has recently emerged as a significant threat to cattle in Africa, Asia, and Europe. Our evidence indicates that *S. calcitrans* is likely to be an important vector species. It also suggests that *Culicoides* biting midges may be a more efficient vector species than previously considered. Furthermore, we have demonstrated for the first time that subclinical and preclinical infected cattle pose only a very limited risk of onward transmission of LSDV to potential vectors. This evidence supports LSD control programs which target clinically affected cattle for rapid removal, rather than complete stamping out of all cattle in an affected herd.

## MATERIALS AND METHODS

### Experimental design.

**(i) Ethical statement, housing, and husbandry.** The experimental study was conducted under the project license P2137C5BC from the UK Home Office according to the Animals (Scientific Procedures) Act 1986. The study was approved by the Pirbright Institute Animal Welfare and Ethical Review Board. Cattle were housed in the primary high-containment animal facilities (biosafety level 3 agriculture) at The Pirbright Institute. The husbandry of the animals during the study was described previously (9).

**(ii) Challenge study and experimental procedures.** Ten Holstein-Friesian male cattle (referred to as calves) were used for the study, which was done in two experimental replicates of five animals each. The median age and weight of the calves were 104 days old and 145 kg in replicate one and 124 days old and 176 kg in replicate two. Eight calves were challenged by intravenous and intradermal inoculation with a suspension of LSDV containing 10^6^ PFU/ml (9). More specifically, 2 ml was intravenously (jugular vein) inoculated and 1 ml was intradermally inoculated in four sites (0.25 ml in each site), two on each side of the neck. The remaining two calves were not challenged and were kept as noninoculated in-contact controls. Calves were randomly assigned to either the control or challenge groups using a random number generator (excluding control calf 1, which was assigned as a control on welfare grounds following diagnosis with shipping fever pneumonia). The calves were kept for 21 days following the challenge; clinical scores were taken daily, and serum, whole blood, and skin biopsy samples (9) were collected over the study period. The nonsteroidal anti-inflammatory drug meloxicam (0.5 mg/kg of body weight) (Metacam 20-mg/ml solution; Boehringer Ingelheim) was used when required on welfare grounds.

**(iii) Insect exposure.** Blood-feeding insects used in the study were Aedes aegypti “Liverpool” strain, Culex quinquefasciatus TPRI line (Tropical Pesticides Research Institute, obtained from the London School of Hygiene and Tropical Medicine, London, UK), *Stomoxys calcitrans* (colony established in 2011 from individuals kindly provided by the Mosquito and Fly Research Unit, USDA Florida), and *Culicoides nubeculosus* (65). All insects were reared at The Pirbright Institute under the following insectary conditions. *Ae. aegypti* was reared in pans of 300 larvae per pan, containing approximately 1 liter of water supplemented with fish food and housed at 28°C, 70% relative humidity (RH), and 12:12 light/dark cycle. *Cx. quinquefasciatus* was reared in pans of 500 to 800 larvae per larval bowl, containing approximately 1.5 liter of water supplemented with ground guinea pig food and maintained at 26°C, 50% RH, and 16:8 light/dark cycle. *S. calcitrans* was reared in approximately 200 eggs per pot and incubated for 12 to 13 days in larval pots containing a ratio of 3:2:1 (powdered grass meal, water, and corn flour) and a tablespoon of yeast. *C. nubeculosus* was reared in approximately 10,000 larvae per pan containing 2 liters of dechlorinated water supplemented with Oxoid broth and dried grass/wheat germ mix. Pots of 800 *Culicoides* pupae were made with males and females and allowed to emerge. Both *S. calcitrans* and *C. nubeculosus* were maintained in insectaries at 27 ± 2°C, 50% RH, with a 16:8 light/dark cycle.

The age and sex composition of the insects at exposure were female and male *C. nubeculosus* between 0 and 2 days posteclosion, female *Cx. quinquefasciatus* and *Ae. aegypti* at 5 to 7 days posteclosion, and male and female *S. calcitrans* at an average of 4 days posteclosion (range, 2 to 7 days). All adult insects were maintained on 10% sucrose and starved 18 to 24 hours before exposure to the calves.

All eight challenged calves, independent of clinical status, were exposed (for between 5 and 20 minutes) to each of the four insect species on days 5, 7, 9, 11, 15, 17, and 19 postchallenge. At each time point, each pot of insects was placed on a cutaneous nodule on a clinical animal and a corresponding area of normal skin on a subclinical animal. The hair of the calf at each feeding site was clipped and/or shaved, and the insects were held in close contact with the skin of the calves in a container covered by mesh. Around 2 hours after exposure, insects were anesthetized under CO_2_, unfed individuals were discarded, and blood-engorged individuals were collected.

For *Ae. aegypti*, *Cx. quinquefasciatus* and *C. nubeculosus* blood engorgement was assessed visually by the presence of blood in the abdominal cavity. However, *S. calcitrans* adults were all collected in a blind manner, and blood engorgement was confirmed by the detection of the bovine *cytochrome b* gene (66) using qPCR. Those individuals negative for *cytochrome b* at collection were removed from the analysis.

Samples from each insect group taken immediately following blood-feeding assessment (dpf 0) were stored at −80°C, and the rest of the insects were maintained for 1, 2, 4 or 8 dpf. After this incubation period, surviving individuals were collected and stored at −80°C after the incubation period. Throughout incubation, all insects were maintained on 10% sucrose solution, except *S. calcitrans* which was maintained with defibrinated horse blood (TCS Biosciences Ltd.) after 2 dpf. All insects were kept in a temperature-controlled room at biocontainment level 3, with a 10:14 light/dark cycle. For the incubation, cardboard/waxed pots containing the insects were placed inside plastic boxes covered by mesh which were kept under a plastic shelter to minimize temperature and humidity fluctuations. Temperature (mean, 24.8°C; range, 22.4°C to 26.4°C) and RH (mean, 35.9%; range, 18.5% to 48.9%) of the room and of the incubation area were recorded approximately every 15 minutes (RF513, Comark Instruments and HOBO UX100-003 Onset).

**(iv) Samples.** Skin biopsy samples were weighed on a calibrated scale (EP613C Explorer Pro; OHAUS) and homogenized in 500 μl high-glucose Dulbecco’s modified Eagle’s medium (41965; Life Technologies) supplemented with 5% fetal bovine serum (Antibody Production Services Ltd., Bedford, UK), 100 U/ml penicillin and 100 μg/ml streptomycin (15140122; Life Technologies), and 2.5 μg/ml amphotericin B (15290026; Life Technologies) in a Lysing Matrix A tube (SKU 116910050-CF; MP Biomedicals) using a portable homogenizer (BeadBug Microtube Homogenizer, D1030; Benchmark Scientific Inc.). Whole insects were homogenized using a TissueLyser (Qiagen, UK) with one or two steel beads of 3 mm (Dejay Distribution, UK) (67) in 200 μl Dulbecco’s phosphate-buffered saline (PBS; 14190094; Life Technologies), supplemented with penicillin-streptomycin and amphotericin B, as described previously. Bovine peripheral blood mononuclear cells (PBMCs) were isolated from 7 ml of whole blood in EDTA diluted in PBS 1:1. The diluted blood was added to a SepMate-50 centrifugation tube (Stemcell Technologies) underlayered with Histopaque-1083 (Sigma-Aldrich). Tubes were centrifuged at 1,500 × *g* for 30 minutes at 20°C with no brake. PBMCs were aspirated from the interface into PBS and then washed three times with PBS at 1,000 × *g* for 10 minutes at 20°C. After the final wash, cells were resuspended in 2 ml of RPMI medium (21875091; Life Technologies) supplemented with 10% fetal bovine serum, and penicillin-streptomycin as above. Blood collected without anticoagulants was allowed to clot and then was spun at 1,000 × *g* to 2,000 × *g* for 10 minutes in a refrigerated centrifuge, and the serum was collected. All samples were stored at −80°C until analyzed.

**(v) Laboratory assays.** Nucleic acid from 200 μl of whole blood, PBMC suspension, or skin homogenate or 100 μl of insect homogenate was extracted in a 96-well plate with the MagMAX CORE nucleic acid purification kit (A32700; Applied Biosystems), using protocol A in a KingFisher Flex magnetic particle processor (Applied Biosystems), and eluted in 50 μl of buffer. qPCR for LSDV ORF074 detection was performed using a modification of the TaqMan assay described by Bowden et al. (68) with the Path-ID qPCR master mix (4388644; Life Technologies). Briefly, a 20-μl reaction mixture was prepared using 5 μl of template, 400 nM each primer, 250 nM the probe, and nuclease-free water to the final volume. Samples were prepared in a 96-well plate and assayed using the Applied Biosystems 7500 Fast real-time PCR system with the following program conditions: 95°C for 10 min and 45 cycles of 95°C for 15 s and 60°C for 60 s. Tissue culture-derived LSDV-positive controls were included in the extraction plates, and the copy number of the LSDV genome was quantified using gBlocks gene fragments (Integrated DNA Technologies) to generate the standard curve. The gBlocks gene fragment included the target sequence of the Bowden assay for detection of LSDV ORF074 (AAATGAAACCAATGGATGGGATACATAGTAAGAAAAATCAGGAAATCTATGAGCCATCCATTTTCCAACTCTATTCCATATACCGTTTT). Bovine blood intake by insects was determined using a SYBR green assay (PowerUp SYBR green master mix, A25779; Life Technologies) for the detection of bovine mitochondrial *cytochrome b* as described by Van Der Saag et al. (66) with some modifications (forward primer, 5′-GTAGACAAAGCAACCCTTAC at 300 nM; reverse primer, 5′-GGAGGAATAGTAGGTGGAC at 500 nM) using the manufacturer cycling conditions for primers with melting temperature (*T_m_*) of >60°C. The assay was performed in a 10-μl reaction mix using 2 μl of the template. This assay was specific for bovine *cytochrome b*, and a melt curve analysis was performed to confirm that only specific amplification occurred. For all qPCR assays, a constant fluorescence threshold was set which produced reproducible quantification cycle (*C_q_*) values for the positive-control samples between runs. A double-antigen ELISA (ID Screen Capripox; IDvet) was used to detect circulating antibodies for LSDV in serum samples following the manufacturer’s protocol and analyzed with the Multiskan FC microplate photometer (Thermo Scientific). Infectious virus titer determinations of PBMC suspension and insect and skin homogenate was performed by viral plaque quantification in Madin-Darby bovine kidney (MDBK) cells.

### Parameter estimation.

Full details of parameter estimation are provided in the Appendix but are briefly described below.

### (i) Probability of transmission from bovine to insect and virus inactivation rate.

The numbers of insects positive for viral DNA after feeding on cattle infected with LSDV were used to estimate the probability of transmission from bovine to insect and the virus inactivation rate. The probability that an insect would be positive when tested is

(1) p=βexp⁡(−γt)

where *β* is the probability of transmission from bovine to insect, *γ* is the virus inactivation rate (i.e., the reciprocal of the mean duration of virus retention), and *t* is the time postfeeding at which the insect was tested. This probability (equation 1) combines the probability that an insect acquired virus (*β*; i.e., the probability of transmission from bovine to insect) and the probability that the insect retained the virus until it was tested at *t* days postfeeding [exp(−*γt*)].

Differences among insect species in the virus inactivation rate and probability of transmission from bovine to insect and in the probability of transmission between subclinical and clinical animals were explored by comparing the fit of models in which these parameters did or did not vary with species or clinical status of the donor cattle. In addition, the dose-response relationship was investigated by allowing the probability of transmission from bovine to insect to depend on the level of viral DNA (in either blood or skin) in the donor animal, so that

(2) $$\log\left(\frac{\beta }{1-\beta }\right)=d_{0}+d_{1}V$$

where *d_0_* and *d_1_* are the dose-response parameters and *V* is the level of viral DNA (log_10_ copies/ml in blood or log_10_ copies/mg in skin) in the donor when the insect fed. The different models were compared using the deviance information criterion (69). The two proxy measures for infectiousness (i.e., level of viral DNA in blood or skin) were compared by computing posterior predictive *P* values for each insect.

### (ii) Probability of transmission from insect to bovine.

Data on transmission of LSDV from insect to bovine were extracted from the published literature (22, 28, 30). In these experiments, batches of insects (of the same species as used in the present study) were allowed to feed on an infected bovine and then to refeed at later time points on a naive recipient. The probability of the recipient becoming infected is

(3) $$q=1-{\left[1-b\beta \exp(-\gamma T)\right]}^{n}$$

where *b* is the probability of transmission from insect to bovine, *β* is the probability of transmission from bovine to insect, *γ* is the virus inactivation rate, *T* is the time interval between feeding on the donor and refeeding on the recipient, and *n* is the number of insects which refed. This probability (equation 3), is the probability that at least one insect (out of the *n* refeeding) transmitted LSDV, where the probability that an individual insect will transmit is the product of the probabilities that it acquired the virus during the initial feed (*β*), retained it until refeeding [exp(−*γT*)], and subsequently transmitted LSDV at refeeding (*b*).

### (iii) Latent and infectious periods in cattle.

Previous estimates for the latent and infectious periods of LSDV (45) were updated using the data on detection of LSDV in blood and skin collected during the present and other recently published studies (28, 32). In addition, the proportion of cattle that develop clinical disease following challenge was estimated using data extracted from the published literature (28, 31, 32, 70, 71) and the present study.

### (iv) Bayesian methods.

Parameters were estimated using Bayesian methods. For all analyses, samples from the joint posterior distribution were generated using an adaptive Metropolis scheme (72), which was modified so that the scaling factor was tuned during burn-in to ensure an acceptance rate of between 20% and 40% for more efficient sampling of the target distribution (73). The adaptive Metropolis schemes were implemented in Matlab (version 2019; The Mathworks Inc.), and the code is available online at https://github.com/SimonGubbins/LSDVAcquisitionAndRetentionByInsects. Two chains were allowed to burn-in and then were run to generate an effective sample size of around 5,000 samples (assessed using the mcmcse package [74] in R, version 3.6.1 [75]). Convergence of the chains was assessed visually and using the Gelman-Rubin statistic provided in the coda package (76) in R (77). Different models for the variation among species in virus inactivation and probability of transmission from bovine to insect (see Appendix) were compared using the deviance information criterion (69).

### Basic reproduction number for LSDV.

The basic reproduction number, denoted by *R*_0_, is the “average number of secondary cases arising from the introduction of a single infected individual into an otherwise susceptible population” (44). The basic reproduction number for LSDV is,

(4) $$R_{0}=\sqrt{\frac{b\beta ma^{2}}{\left(\mu +\gamma \right)}\left(p_{C}\frac{1}{r_{C}}+(1-p_{C})\rho \frac{1}{r_{S}}\right)},$$

where *b* is the probability of transmission from insect to bovine; *β* is the probability of transmission from bovine to insect; *ρ* is the relative risk of transmission from a subclinical compared with a clinical bovine; *γ* is the virus inactivation rate; *p_C_* is the proportion of cattle that develop clinical disease; and 1/*r_C_* and 1/*r_S_* are the mean durations of infectiousness for clinical and subclinical animals, respectively, all of which were estimated in the present study. Additionally, *a*, *m*, and *μ* are the biting rate, vector-to-host ratio, and vector mortality rate, respectively. The formal derivation of the expression for *R*_0_ in equation 4 is given in the Appendix.

Replicated Latin hypercube sampling was used to compute the median and 95% prediction interval for *R*_0_ for each insect species (45). Parameters were sampled either from their marginal posterior distributions derived in the present study (*b*, *β*, *ρ*, *γ*, *p_C_*_,_ 1/*r_C_*, and 1/*r_S_*; see Table 1 and Appendix) or uniformly from plausible ranges (*a*, *m*, and *μ*; see Appendix). The mean duration of infection for clinical animals (1/*r_C_*) is based on the detection of virus or viral DNA in skin, while that for subclinical animals (1/*r_S_*) is based on detection of viral DNA in blood (see Appendix).

### Data availability.

We declare that the main data supporting the findings of this study are available within the article and in Data Set S1. The code and the data used are available online for readers to access with no restriction at https://github.com/SimonGubbins/LSDVAcquisitionAndRetentionByInsects.

## Supplementary Material

Supplemental file 1

## APPENDIX. MODELING THE TRANSMISSION OF LUMPY SKIN DISEASE VIRUS BY HEMATOPHAGUS INSECTS

### Probability of transmission from bovine to insect and virus inactivation rate.

For each insect, the clinical status of the donor calf (i.e., clinical or subclinical), the number of days postfeeding at which the insect was tested, and whether the insect was positive for lumpy skin disease virus (LSDV) DNA (defined as any viral DNA detected) were used to estimate the probability of transmission from bovine to insect and the virus inactivation rate.

The likelihood for the data is given by

(A.1) $$L=\prod_{i}p_{i}^{\delta_{i}}{\left(1-p_{i}\right)}^{1-\delta_{i}}$$

where *δ_i_* is an indicator for whether insect *i* was negative (*δ_i_* = 0) or positive (*δ_i_* = 1) for viral DNA when tested for LSDV, and *p_i_* is the probability that insect *i* was positive when tested. If insect *i* fed on a clinically affected animal, this is given by

(A.2) $$p_{i}=\beta_{s_{i}}\exp(-\gamma_{s_{i}}t_{i})$$

while if it fed on a subclinical donor, it is given by

(A.3) $$p_{i}=\rho_{s_{i}}\beta_{s_{i}}\exp(-\gamma_{s_{i}}t_{i})$$

Equations A.2 and A.3 combine (i) the species-specific probability that an insect became infected (*β_s_* or *ρ_s_β_s_*, depending on the status of the donor calf, where *β_s_* is the probability of transmission from a clinical bovine to an insect and *ρ_s_* is the relative risk of transmission from a subclinical donor compared to a clinical one); and (ii) the probability that the insect was still positive when it was tested at *t_i_* days postfeeding [exp(−*γ_s_t_i_*)], where *γ_s_* is the species-specific virus inactivation rate.

Differences among insect species in the virus inactivation rate, probability of transmission from bovine to insect, and relative risk of transmission were incorporated in the model by assuming hierarchical structure in the parameters, so that

(A.4) $$\begin{array}{l} \gamma_{s}∼\text{Gamma}(k_{\gamma },\mu_{\gamma }),\\ \beta_{s}∼\text{Beta}(a_{\beta },b_{\beta }),\\ \rho_{s}∼\text{Gamma}(k_{\rho },\mu_{\rho }) \end{array}$$

where *k_γ_* and *k_ρ_* are shape parameters, *μ_γ_* and *μ_ρ_* are means for a gamma distribution, and *a_β_* and *b_β_* are the parameters for a beta distribution. If and how the parameters varied among species were explored by considering the fit of the model when no, one, two, or three of the parameters varied among the insect species. In total, 12 models were considered (see Table A1).

**TABLE A1:** Comparison of models for the acquisition and retention of LSDV by biting insects^*a*^

Dose-independent model				Dose-response model				
Inactivation rate	Probability of transmission	Relative risk of transmission	DIC^*b*^	Inactivation rate	Intercept	Slope	DIC^*b*^	
							Blood^*c*^	Skin^*c*^
Common	Common	No difference	1,335.3	Common	Common	Zero	1,335.3	1,335.3
Varies	Common	No difference	1,334.6	Common	Common	Common	874.2	684.6
Common	Varies	No difference	1,338.6	Common	Varies	Common	871.1	682.0
Varies	Varies	No difference	1,340.2	Common	Common	Varies	869.0	681.4
Common	Common	Common	1,025.5	Common	Varies	Varies	868.9	680.7
Varies	Common	Common	**1,021.0**	Varies	Common	Zero	1,334.6	1,334.6
Common	Varies	Common	1,026.8	Varies	Common	Common	**863.8**	**675.1**
Varies	Varies	Common	1,025.9	Varies	Varies	Common	864.0	676.5
Common	Common	Varies	1,025.3	Varies	Common	Varies	863.9	677.1
Varies	Common	Varies	1,019.5^*d*^	Varies	Varies	Varies	863.9	677.4
Common	Varies	Varies	1,025.5
Varies	Varies	Varies	1,024.5

a Common, parameter common to all insect species; varies, parameter varies among insect species; no difference, risk of transmission does not differ between clinical and subclinical animals; zero, parameter fixed at zero.

b A model with a lower DIC is preferred to one with higher DIC. DIC, deviance information criterion. The model with its DIC shown in bold is the one preferred.

c Level of viral DNA in blood or skin used as the proxy measure for infectiousness.

d Although this model has a smaller DIC than the one shown in bold, the difference is less than two and the simpler model was preferred as it has the smaller number of parameters.

Hierarchical priors were used for those parameters that differed among species, and exponential priors (with mean 1) were used for the higher-order parameters in the hierarchical distributions. Where the parameters were common to all species, an exponential prior (with mean 100) was used for *γ* or *ρ*, and a uniform prior (with range [0,1]) was used for *β*.

Samples from the joint posterior density were generated using an adaptive Metropolis scheme (72), modified so that the scaling factor was tuned during burn-in to ensure an acceptance rate of between 20% and 40% for more efficient sampling of the target distribution (73). Two chains of 6,000,000 iterations were run, with the first 1,000,000 iterations discarded to allow for burn-in of the chain. The chains were then thinned (taking every 500th sample) to reduce autocorrelation among the samples. The adaptive Metropolis scheme was implemented in Matlab (version R2019b; The Mathworks Inc.) and the code is available online at https://github.com/SimonGubbins/LSDVAcquisitionAndRetentionByInsects. Convergence of the scheme was assessed visually and by examining the Gelman-Rubin statistic provided in the coda package (76) in R (version 3.6.1) (75). Different models for the variation among species in virus inactivation and probability of transmission from bovine to insect were compared using the deviance information criterion (DIC) (69).

### Dose-response relationship.

For each insect, the level of lumpy skin disease viral DNA in blood (log_10_ copies/ml) or skin (log_10_ copies/mg) for the calf at the time when the insect fed, the number of days postfeeding at which the insect was tested, and whether or not the insect was positive for LSDV DNA (defined as any viral DNA detected) were used to investigate the dose-response relationship for the probability of transmission. Only data for insects which fed on the clinically affected donors were included in this analysis.

The likelihood for the data is given by equation A.1, but the probability that insect *i* was positive is given by

(A.5) $$p_{i}=\beta_{i}\exp(-\gamma_{s_{i}}t_{i})$$

where *β_i_* is the probability of transmission from bovine to insect for insect *i*, *γ_s_* is the virus inactivation rate for species *s*, and *t_i_* is the number of days postfeeding at which insect *i* was tested. The dose-response relationship between the probability of transmission from bovine to insect (i.e., *β_i_*) and level of viral DNA was described by

(A.6) $$\log\left(\frac{\beta_{i}}{1-\beta_{i}}\right)=a_{s_{i}}+b_{s_{i}}V_{c_{i}}(t_{f}^{\left(i\right)})$$

where *a_s_* and *b_s_* are (insect) species-specific dose-response parameters and *V_j_*(*t*) is the level of viral DNA in calf *j* at *t* days postinfection, *c_i_* is the calf on which insect *i* fed, and $t_{f}^{\left(i\right)}$ is the time of feeding for insect *i*.

Variation among insect species in virus inactivation rates (*γ*) and dose response (*a* and *b*) was incorporated in the model by assuming hierarchical structure in the parameters, so that

(A.7) $$\begin{array}{l} \gamma_{s}∼\text{Gamma}(s_{\gamma },\mu_{\gamma }),\\ a_{s}∼\text{Normal}(\mu_{a},\sigma_{a}^{2}),\\ b_{s}∼\text{Normal}(\mu_{b},\sigma_{b}^{2}) \end{array}$$

where *μ_γ_*, *μ_a_*, and *μ_b_* are the distribution means; *s_γ_* is the shape parameter for the gamma distribution; and *σ_a_* and *σ_b_* are the standard deviations for the normal distributions. The following five possibilities were considered for the parameters relating titer to the probability of transmission: (i) independent of titer (i.e., *a* common to all species and *b *= 0), (ii) *a* and *b* were common to all species, (iii) *a* varied among species and *b* was common to all species, (iv) *a* was common to all species and *b* varied among species, and (v) *a* and *b* varied among species. In addition, two possibilities were considered for the virus inactivation rate, namely, (i) common to all species or (ii) varies among species. Consequently, 10 models were considered (Table A1).

Where the parameters were common to all species, an exponential prior (with mean 100) was used for *γ* and normal priors (with mean 0 and standard deviation 10) were used for *a* or *b*. Hierarchical priors were used for those parameters that differed among species. For the higher-order parameters in the hierarchical distributions, an exponential prior (with mean 1) was used for *μ_γ_* and normal priors (with mean 0 and standard deviation 10) were used for *μ_a_* and *μ_b_*, while exponential priors with mean 1 were used for *s_γ_*, *σ_a_*, and *σ_b_*.

Samples from the joint posterior density were generated using an adaptive Metropolis scheme as described in the “Probability of transmission from bovine to insect and virus inactivation rate” section. Two chains of 6,000,000 iterations were run, with the first 1,000,000 iterations discarded to allow for burn-in of the chain. The chains were then thinned (taking every 500th sample) to reduce autocorrelation among the samples.

The different models for the relationship between titer and infection were compared using the deviance information criterion (69). The two proxy measures for infectiousness (i.e., level of viral DNA in blood or skin) were compared by computing posterior predictive *P* values for each insect. Specifically, the joint posterior distribution for the model was sampled and the probability that each insect was positive for viral DNA computed. Whether or not the insect was positive when tested was then simulated, and the observed outcomes were compared to the simulated ones. This procedure was repeated multiple times, and the proportion of samples for which the observed and simulated outcomes matched was computed (i.e., the posterior predictive *P* value).

### Probability of transmission from insect to bovine.

Previous studies have considered the transmission of LSDV from insect to bovine for the four insect species used in the present study (22, 28, 30). The results of these earlier studies were analyzed in light of the results from the present study to estimate the probability of transmission from insect to bovine for each species.

### (i) Aedes aegypti and Culex quinquefasciatus.

A batch of insects was fed on a clinically affected bovine (*Ae. aegypti*) or a blood-virus mix via a membrane (*Cx. quinquefasciatus*) and, subsequently, allowed to refeed on a naive bovine, with each recipient fed on only once (22, 30). The likelihood for the data for each species is

(A.8) $$L=\prod_{j}q_{j}^{I_{j}}{\left(1-q_{j}\right)}^{1-I_{j}}$$

where *I_j_* is a variable indicating whether (*I_j_* = 1) or not (*I_j_* = 0) transmission occurred when insects were allowed to refeed on naive animal *j* at time *t_j_* post-initial feed and

(A.9) $$q_{j}=1-{\left[1-b\beta \exp(-\gamma t_{j})\right]}^{n_{j}}$$

is the probability of transmission for the challenge. The probability (equation A.9) is the probability that at least one insect (out of the *n_j_* refeeding) transmitted LSDV, where the probability that an individual insect will transmit is the product of the probabilities that it became infected during the initial feed (*β*), was still infected at refeeding [exp(−*γt_j_*)], and subsequently transmitted LSDV at refeeding (*b*). The marginal posterior densities for each species obtained in section “Probability of transmission from bovine to insect and virus inactivation rate” were used as informative priors for *β* and *γ*, while a noninformative prior (uniform with range [0,1]) was used for *b*.

Samples from the joint posterior density were generated using an adaptive Metropolis scheme similar to that described in section “Probability of transmission from bovine to insect and virus inactivation rate.” Two chains of 75,000 iterations were run, with the first 25,000 iterations discarded to allow for burn-in of the chain. The chains were then thinned (taking every 5th sample) to reduce autocorrelation among the samples.

### (ii) Culicoides nubeculosus.

The experimental design for *C. nubeculosus* (30) was the same that as described above for *Ae. aegypti*. However, the number of insects refeeding was not reported; yet, this value is required when estimating the probability of transmission from insect to bovine (see equation A.9). Accordingly, the numbers of insects refeeding in each batch were included in a data augmentation step in the Bayesian framework (i.e., as nuisance parameters). In this case, the number of insects refeeding was drawn from a binomial distribution for this species, so that

(A.10) $$n_{j}∼\text{Binomial}(N_{j},1-\exp(-at_{j}))$$

where *N_j_* is the number of insects used for challenge *j* (assumed to be 100, which is approximately the maximum number of insects fed), *a* is the biting rate, and *t_j_* is the time between feeding on the donor and refeeding on the recipient. A uniform prior with range (0, 0.4) was used for *a* (based on the duration of the gonotrophic cycle of *Culicoides* biting midges [78–80]).

Samples from the joint posterior density were generated using an adaptive Metropolis scheme similar to that described in section “Probability of transmission from bovine to insect and virus inactivation rate.” Two chains of 600,000 iterations were run, with the first 100,000 iterations discarded to allow for burn-in of the chain. The chains were then thinned (taking every 50th sample) to reduce autocorrelation among the samples.

### (iii) *Stomoxys calcitrans.*

Two studies have examined transmission of LSDV by *S. calcitrans* (28, 30). The same experimental design described above for *C. nubeculosus* was used by Chihota and coauthors (30), so the same likelihood applies for these data (i.e., including the data augmentation step). In the experiments conducted by Sohier and coauthors (28), batches of insects were allowed to feed on an infected donor for 10 minutes and, 1 hour later, were allowed to refeed on a naive recipient for 10 minutes. For each batch, this procedure was repeated one, two, or three times at daily intervals.

The probability that an animal became infected (with onset of viremia on day *T* post initial feed) is given by

(A.11) $$p_{\mathrm{POS}}=1-\prod_{j}\left(1-p_{j}\right)$$

where

(A.12) $$p_{j}=\sum_{k}\left\{q_{\mathrm{jk}}\prod_{i=1}^{k-1}\left(1-q_{\mathrm{ji}}\right)\right\}\int_{T-t_{\mathrm{jk}}-1}^{T-t_{\mathrm{jk}}}f(\tau )\text{d}\tau$$

is the probability of transmission for batch of insects *j*. Here, *q_jk_* is the probability of transmission during the *k*th feed of batch *j* (on day *t_jk_*), and *f* is the probability density function for the gamma-distributed latent period for LSDV (with mean 1/*r_E_* and shape parameter *k_E_*). The first term in the summation (in braces) is the probability the animal became infected on the *k*th feed (and was not infected in any of the previous feeds). The second term (the integral) is the probability that, if the animal became infected on the *k*th feed, the onset of viremia would occur on day *T* after the initial feed.

The probability of transmission during feed *k* for batch of insects *j* is given by

(A.13) $$q_{\mathrm{jk}}=1-{\left(1-b\sum_{i=1}^{k}\beta \exp(-\gamma (t_{\mathrm{ji}}-t_{j1}+\delta ))\right)}^{n_{\mathrm{jk}}}$$

This is the probability that at least one insect (out of the *n_jk_* refeeding) transmitted LSDV. The probability that an individual insect will transmit is the probability that it became infected during the any of preceding feeds on the infected donor and was still infected at refeeding {*β*exp[−γ(*t_ji_*-*t_j_*_1_ + *δ*)], where *δ* is the time interval between feeding on the donor and refeeding on the recipient}, multiplied by the probability that it transmitted LSDV at refeeding (*b*). If the donor animal was subclinical, the *β* in this expression is replaced by *ρβ*. Because the numbers of insects refeeding were not known, they were included as nuisance parameters (cf. *C. nubeculosus* above), with

(A.14) $$n_{\mathrm{jk}}∼\text{Binomial}\left[N_{j},1-\exp(-a\delta )\right]$$

where *N_j_* is the number of insects in batch *j* and *a* is the biting rate.

The probability that an animal remained uninfected is given by the probability that transmission did not occur at any of the feeds for any of the batches of insects, that is

(A.15) $$p_{\mathrm{NEG}}=\prod_{j}\prod_{k}\left(1-q_{\mathrm{jk}}\right)$$

where *q_jk_* is as defined in equation A.13.

The likelihood for the Sohier data is

(A.16) $$L=\prod_{a}p_{\mathrm{NEG}}^{1-I_{a}}p_{\mathrm{POS}}^{I_{a}}$$

where *δ_a_* is a variable indicating whether (*I_a_* = 1) or not (*I_a_* = 0) transmission occurred to animal *a*, and *p_POS_* and *p_NEG_* are the probabilities that an animal became infected or remained uninfected (given by equations A.11 and A.15), respectively. The full likelihood for the *S. calcitrans* data is the product of that for the Chihota data [given by equation (A.8)] and that for the Sohier data [given by equation (A.16)].

The marginal posterior densities for *β*, *ρ*, and *γ* derived for *S. calcitrans* in section “Probability of transmission from bovine to insect and virus inactivation rate” were used as informative priors for these parameters. A noninformative prior (uniform with range [0, 1]) was used for *b*. A gamma prior (with mean 2.26 and shape parameter 2.27) was used for the biting rate *a*, based on an interval between blood meals of 4 to 72 hours (51, 81). The marginal posterior densities obtained from an analysis of previous challenge experiments and the present study (see section “Latent and infectious periods for lumpy skin disease virus in cattle” for details) were used as informative priors for the mean and shape parameter for the latent period distribution (Table A2).

**TABLE A2:** Parameters for the duration of latent and infectious periods for lumpy skin disease virus in cattle

Proxy measure of infectiousness	Shape parameter (*k_i_*)^*a*^	Mean (1/*r_i_*) (days)^*a*^
Latent period (days)
Skin lesions^*b*^	38.0 (10.9, 103.3)	7.6 (6.6, 8.9)
Viremia (PCR)
Clinically affected animals	3.8 (2.0, 6.9)	5.4 (4.2, 7.1)
Subclinical animals	3.9 (1.4, 9.1)	5.5 (3.9, 8.3)
Infectious period (days)
Skin biopsy samples^*b*^	11.4 (2.8, 38.2)	23.6 (17.1, 38.0)
Viremia (PCR)
Clinically affected animals	3.4 (1.3, 7.6)	19.0 (13.7, 30.4)
Subclinical animals	2.5 (0.9, 5.7)	15.2 (10.2, 27.2)

a Posterior median (95% credible interval).

b Clinically affected animals only.

Samples from the joint posterior density were generated using an adaptive Metropolis scheme similar to that described in section “Probability of transmission from bovine to insect and virus inactivation rate” above. Two chains of 11,000,000 iterations were run, with the first 1,000,000 iterations discarded to allow for burn-in of the chain. The chains were then thinned (taking every 1000th sample) to reduce autocorrelation among the samples.

### Latent and infectious periods for lumpy skin disease virus in cattle.

The mean (1/*r_i_*) and shape parameter (*k_i_*) for the gamma-distributed latent (*i *=* E*) and infectious (*i *=* I*) periods for LSDV in clinically affected cattle have been estimated previously (Gubbins 2019) using data from experimental infections of cattle (22, 31, 71). These estimates were updated (Table A2) using the outcome of the present study for two proxy measures of infectiousness, namely, detection of viral DNA in blood and detection of virus or viral DNA in skin biopsy samples. This was done using methods described previously (45), but with the marginal posterior densities from the earlier analysis providing informative priors for the mean and shape parameters.

To estimate the corresponding parameters for subclinical cattle (Table A2), detection of viral DNA in blood in experimentally infected cattle as reported in published data (28, 31, 32) and the present study was used as a proxy measure of infectiousness (as the animals did not have skin lesions). In this case, methods described previously (45) were used, including noninformative priors.

### Proportion of cattle developing generalized disease.

The proportion of cattle developing generalized disease (*p_C_*) was estimated based on the numbers of cattle that developed generalized disease following challenge in published experiments, as follows: 5 out of 14, 4 out of 25, and 8 out of 11 (70); 3 out of 6 (32); 2 out of 6 (31); 6 out of 7 (71); 1 out of 4, 3 out of 5, and 4 out of 5 (28); and 3 out of 8 (present study).

Parameters were estimated in a Bayesian framework. The likelihood for the data is

$$L=\prod_{i}\left(\begin{array}{c} N_{i}\\ C_{i} \end{array}\right)p_{i}^{C_{i}}{\left(1-p_{i}\right)}^{N_{i}-C_{i}}$$

where *C_i_* and *N_i_* are the number of cattle developing generalized disease and the number of challenged cattle, respectively, and *p_i_* is the expected proportion of cattle developing generalized disease in experiment *i*. To allow the *p_i_*s to vary among experiments, a hierarchical structure was assumed, such that

$$p_{i}∼\text{Beta}(\alpha_{p},\beta_{p})$$

where *α_p_* and *β_p_* are hierarchical parameters. Exponential priors (with mean 100) were assumed for *α_p_* and *β_p_*. Samples from the joint posterior distribution were generated by Markov chain-Monte Carlo methods implemented in OpenBUGS (version 3.2.3). Two chains each of 120,000 samples were run, with the first 20,000 iterations discarded to allow for burn-in. The chains were then thinned (selecting every 20th sample) to reduce autocorrelation. The posterior median (95% confidence interval [CI]) for *α_p_* and *β_p_* were 38.5 (3.4 to 196.4) and 49.0 (3.6 to 256.7), respectively, with a corresponding posterior median for *p_C_* of 0.44 (95% CI, 0.34 to 0.55).

A simpler model in which the proportion of cattle developing generalized disease was common to all experiments was also considered. However, this yielded a poorer fit to the data, as judged by the deviance information criterion of 44.0 (*p* varying) versus 48.0 (*p* common).

### Basic reproduction number for lumpy skin disease virus in cattle.

The basic reproduction number *R*_0_ is calculated as the dominant eigenvalue *r*(*K*) of the next-generation operator *K* (82, 83). For lumpy skin disease virus, the next-generation operator is a matrix, K, whose elements (*K_ij_*) are the expected number of infections of type *i* (either clinical cattle [*C*], subclinical cattle [*S*] or vectors [*V*]) arising from a single infected individual of type *j* (cf. reference 45). In this case, K can be written as,

(A.17) $$\text{K}=\left(\begin{array}{ccc} K_{\mathrm{CC}} & K_{\mathrm{CS}} & K_{\mathrm{VC}}\\ K_{\mathrm{SC}} & K_{\mathrm{SS}} & K_{\mathrm{VS}}\\ K_{\mathrm{VC}} & K_{\mathrm{SV}} & K_{\mathrm{VV}} \end{array}\right)$$

Some elements of K are straightforward to derive; there is assumed to be no direct transmission between hosts or vectors (i.e., *K_CC_* = *K_CS_* = *K_SC_* = *K_SS_* = *K_VV_* = 0). The remaining elements, representing transmission from vector to host (*K_CV_* and *K_SV_*) or host to vector (*K_VC_* and *K_VS_*), are computed as follows.

### (i) Transmission from vector to host.

After an insect feeds on an infected bovine, it remains infected (and infectious) until the virus becomes inactivated or the insect dies, a period which lasts on average 1/(*γ+μ*) days, where *γ* is the virus inactivation rate and *μ* is the vector mortality rate. During this time, the infected insect will bite susceptible cattle *a* times per day and a proportion, *b*, of these bites will result in a newly infected animal. Of these, a proportion, *p_C_*, will go on to develop generalized disease, while the remainder, *p_S_* = 1 − *p_C_*, will remain subclinical. Hence, the expected number of infected cattle of each type per infected insect (*K_iV_*, *i *=* C*,*S*) is given by

(A.18) $$K_{\mathrm{iV}}=\frac{\mathrm{ba}}{\gamma +\mu }p_{i}.$$

### (ii) Transmission from host to vector.

An infected bovine remains infectious for 1/*r_i_* days. During this time, it will be bitten by susceptible insects on average *m *×* a* times per day, a proportion, *β_i_* (where *β_C_* = *β* and *β_S_* = *ρβ*, where *β* is the probability of transmission from bovine to insect for clinically affected animals, and *ρ* is the relative risk of transmission from a subclinical compared with a clinical bovine), of which will result in a newly infected vector. Consequently, the expected number of infected insects per infected bovine (*K_Vi_*) is given by

(A.19) $$K_{\mathrm{Vi}}=\frac{\beta_{i}ma}{r_{i}}$$

Some linear algebra shows that the dominant eigenvalue of K (i.e., *R*_0_) is

(A.20) $$R_{0}=\sqrt{K_{\mathrm{CV}}K_{\mathrm{VC}}+K_{\mathrm{SV}}K_{\mathrm{VS}}}$$

which, on substituting the expressions in equation A.18 and A.19 becomes that given in equation 4). Parameter estimates used to compute *R*_0_ are provided in Table 1, A2, and A3.

**TABLE A3:** Life history parameters for four putative insect vectors of lumpy skin disease virus^*a*^

Species	Biting rate (/day)	Vector-host ratio	Mortality rate (/day)
*Ae. aegypti*	0.18–0.23	0–80	0.07–0.2
*Cx. quinquefasciatus*	0.08–0.25	0–80	0.07–0.84
*C. nubeculosus*	0–0.4	0–5,000	0.1–0.5
*S. calcitrans*	0.33–6	30–145	0.04–0.11

a Data from reference 45, distributed under a Creative Commons Attribution License.
